# Pyrazolylpyrimidinamines Decorated via Petasis Reaction as Small-Molecule Activators of the RNA-Degrading Ribonuclease IRE1α

**DOI:** 10.1021/acsbiomedchemau.5c00161

**Published:** 2026-02-27

**Authors:** Amrutha K. Avathan Veettil, Yang Liu, Leon Wagner, Oguz Hastürk, Nguyen Song Thu Huynh, Giorgia Mancino, Maria Beerbaum, Peng Wu

**Affiliations:** † Chemical Genomics Centre, 28268 Max Planck Institute of Molecular Physiology, Dortmund 44227, Germany; ‡ Department of Chemical Biology, Max Planck Institute of Molecular Physiology, Dortmund 44227, Germany; § Department of Chemistry and Chemical Biology, 14311 TU Dortmund University, Dortmund 44227, Germany; ∥ Drug Discovery Hub Dortmund (DDHD), Zentrum für Integrierte Wirkstoffforschung (ZIW), Dortmund 44227, Germany

**Keywords:** Petasis reaction, ribonuclease, RNA degradation, small-molecule activator, pyrimidinamine

## Abstract

The multicomponent Petasis boron-Mannich reaction (PR) enables the generation of functionalized amines that are of biological interest. Here, we demonstrated that a series of pyrazolylpyrimidinamines decorated via PR are new small-molecule activators of the dual kinase and ribonuclease RNA-degrading protein inositol-requiring enzyme 1α (IRE1α), which is an essential effector in the unfolded protein response associated with many human diseases. Compound SH4 was identified via a FRET assay and showed potent activity in activating the IRE1α ribonuclease (RNase) activity, inducing increased *XBP1* mRNA splicing, and inducing *Bloc1s1* mRNA degradation. Based on a binding mode analysis, the following series of PR-decorated functionalized amines was further probed as IRE1α RNase activators. One PR-derived compound, AK177, showed nanomolar activating potency in biochemical assays but minimal activities in cellular evaluations. Overall, we present here a series of pyrazolylpyrimidinamines as new small-molecule activators of the IRE1α RNase activity, which served as the first examples of applying PR in accessing bioactive compounds targeting the kinase domain of a ribonuclease involved in mRNA cleavage and splicing.

## Graphical abstract

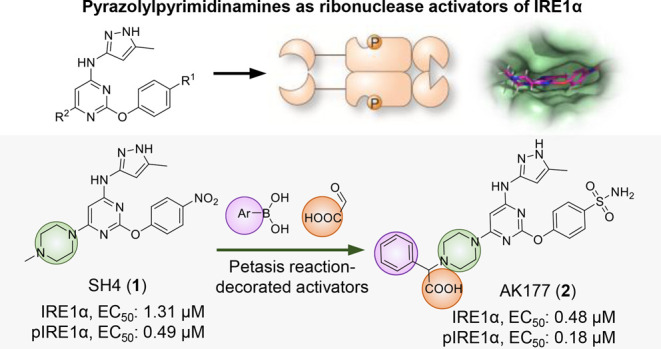

## Introduction

Multicomponent reactions are efficient synthetic methods to access small molecules of new scaffolds covering new chemical space that are of interest for medicinal chemistry and drug discovery purposes.^1^ The Petasis boron-Mannich reaction (PR) is a complexity-generating multicomponent reaction that enables the functionalization of bioactive amines with a boronic acid and a carbonyl component (Figure 1A).^2,3^ PR features many merits, such as a metal-free transformation that does not need the presence of expensive or environmentally damaging metals and other catalysts. Recent examples in the field include the late-stage functionalization of bioactive aliphatic amines assisted by light irradiation,^4^ rapid generation of complex pharmaceutically relevant alkyl amines,^5^ peptide stapling enabled by tryptophan-mediated PR,^6^ total synthesis of natural products—strempeliopidines,^7^ and generation of polycyclic scaffolds with a high content of sp^3^-hybridized carbon atoms.^8^

**Figure 1:**
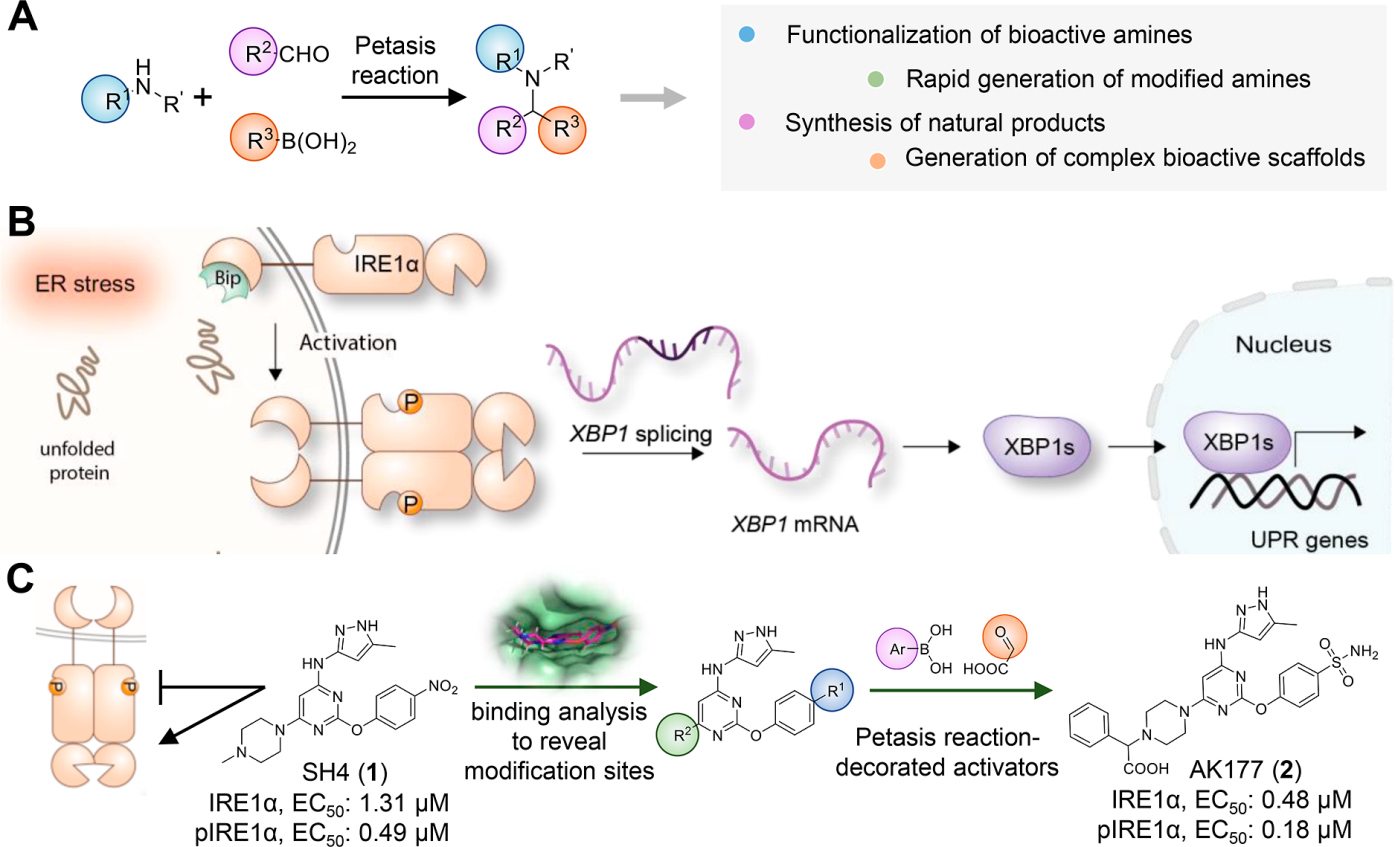
Pyrazolylpyrimidinamines decorated by Petasis reaction (PR) as new IRE1α activators. (A) Three-component PR of amine, aldehyde, and boronic acid and application of PR in medicinal chemistry and drug discovery to yield bioactive small molecules. (B) A brief illustration of the inositol-requiring enzyme 1 alpha (IRE1α)-mediated unfolded protein response (UPR) pathway leading to *XBP1* RNA splicing. (C) Results reported in this work in applying PR to obtain functionalized piperazines as new IRE1α activators.

Despite the encouraging progress achieved in applying PR to access bioactive molecules in general, one limitation in applying PR in medicinal chemistry and drug discovery is the lack of target characterization for such bioactive PR-decorated functionalized amine molecules and the insufficient mechanistic study of PR products. We herein report the first example of PR-generated amines that target the kinase domain of a ribonuclease involved in mRNA cleaving and splicing, specifically, as new small-molecule activators of the ribonuclease—inositol-requiring enzyme 1 alpha (IRE1α), which is the most evolutionarily conserved component of the unfolded protein response (UPR) pathway.^9^ IRE1α is one of the two paralogues of IRE1, being a multidomain sensor protein, with an *N*-terminal ER luminal domain and a *C*-terminal cytosolic domain, the latter of which holds dual kinase and endoribonuclease (RNase) activities.^10–12^ In IRE1α-mediated UPR pathways, the sensing of unfolded protein by the luminal domain results in self-association, autophosphorylation, and higher-order assembly, leading to unconventional splicing of the mRNA encoding X-box binding protein 1 (XBP1) and translation of the spliced isoform XBP 1s (Figure 1B).^13,14^ Additionally, IRE1α can directly degrade cytosolic RNA species via regulated IRE1-dependent decay (RIDD).^15^ In situations where the ER stress cannot be contained via adaptive UPR, it will lead to apoptosis. Dysregulation of the UPR is implicated in many diseases.^16^ Notably, the IRE1α-*XBP1* branch of the UPR is linked to a variety of cancers.^17^ Hence, modulation of the IRE1α activity via small molecules is an attractive therapeutic strategy, which includes small molecules that inhibit the IRE1α RNase to impair the adaptability of tumor cells to the challenging tumor microenvironment and small molecules that activate or hyperactivate IRE1α RNase to initiate cell death.^18–21^

In our continued efforts to discover new small molecules targeting RNA-binding, RNA-modifying, and RNA-cleaving proteins,^22–25^ we identified potent and selective IRE1α-targeting inhibitors based on an indole scaffold, which revealed an inhibition mode via binding to the IRE1α kinase domain and inhibited the IRE1α RNase activity by disrupting the RNase domain dimerization.^26^ Apart from the inhibitors, we identified a pyrazolylpyrimidinamine-based molecule that showed IRE1α RNase activating potency instead of inhibition. Structure-based analysis revealed a potential for further structural modification to enhance the activity of such activators based on the pyrazolylpyrimidinamine scaffold. Therefore, we reported herein our efforts in decorating the pyrazolylpyrimidinamine via three-component PR to obtain functionalized piperazines as new IRE1α activators toward both phosphorylated and unphosphorylated IRE1α (Figure 1C).

## Results and Discussion

In search of new IRE1α-targeting scaffolds, we evaluated an in-house collection of small-molecule kinase inhibitors in a FRET assay using unphosphorylated IRE1α (residues 547-977) and an *XBP1* RNA hairpin probe labeled with FAM and a black hole quencher (BHQ) (5′-FAM-CAUGUCCGCAGCGCA UG-3′ BHQ1). Among the small molecules tested, the aminopyrazole compound SH4 (1) showed potent single-digit micromolar activation potency toward unphosphorylated IRE1α in the FRET assay measured by the increase of FAM fluorescence via cleavage of the RNA probe (Figure 2A). SH4 showed EC_50_ of 1.31 μM and 0.49 μM toward unphosphorylated and phosphorylated IRE1α, respectively (Figure 2B,C). Furthermore, SH4 showed concentration-dependent cleavage of the *XBP1* mRNA probe in a gel-based cleavage assay (Figure 2D,E). In the microscale thermophoresis (MST), it showed a binding affinity ranging between 167 and 730 nM toward IRE1α (Figure S1). To probe the activation mechanism of SH4, we then performed the cross-linking assay measuring the dimerization of IRE1α using the Refeyn mass photometry. Disuccinimidyl suberate (DSS, Figure 2F) and the reported allosteric IRE1α activator APY29 (Figure 2G) were tested together with SH4 in the cross-linking assay, the results of which demonstrated that both APY29 and SH4 led to increased IRE1α dimerization (Figures 2H,S2, and S3).

**Figure 2:**
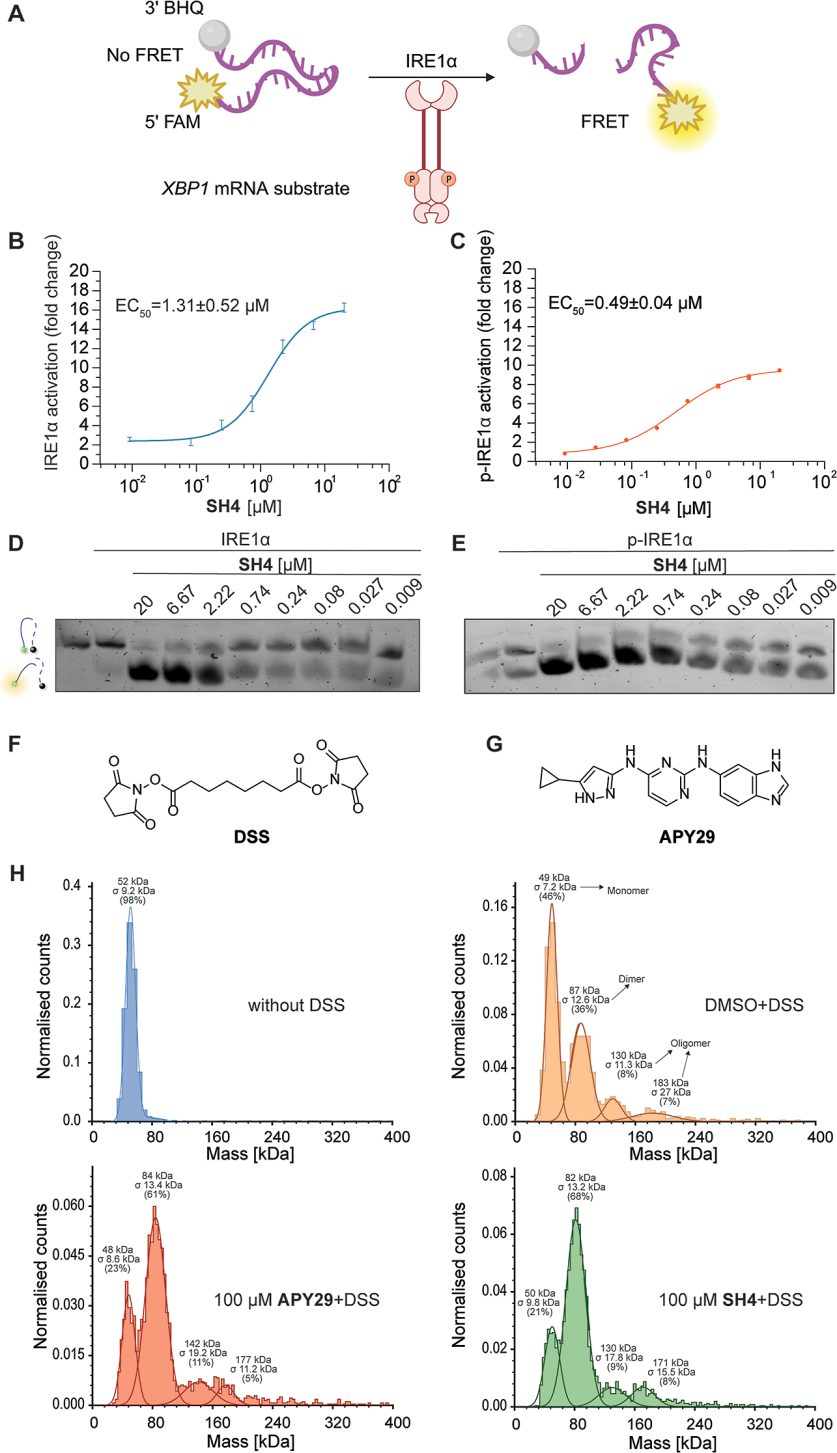
Identification of the pyrazolylpyrimidinamine SH4 as an IRE1α activator. (A) Schematic representation of the FRET assay using an *XBP1* RNA probe labeled with FAM and BHQ led to the identification of SH4 as an IRE1α activator. (B,C) SH4 activated unphosphorylated and phosphorylated IRE1α in the FRET assay; data are presented as mean ± SD, *n* = 3. (D,E) SH4 concentration-dependently activated unphosphorylated and phosphorylated IRE1α in the gel-based cleavage assay. (F) Structure of DSS used in the cross-linking assay. (G) The reported compound APY29 used in the cross-linking assay. (H) SH4 activates IRE1α activity by promoting the dimerization, measured in the Refeyn mass photometry (the reported activator APY29 was used as a comparison).

To develop more potent and selective IRE1α activators, we performed molecular docking based on the complex structure between another reported activator APY29 and IRE1.^19^ Based on the docking result, SH4 likely utilizes the allosteric RNase activation mechanism of APY29 by functioning as a kinase domain binder (Figure 3A and B). The optimal docking conformation revealed a similar binding mode to that of APY29 at the kinase active site (Figure 3C–E), in which the pyrazolylpyrimidinamine moiety interacts with the hinge region. The 4-nitrophenoxy substituent at the pyrimidine-2-position of SH4 mimicked the benzoimidazolamine substituent at the 2-pyrimidine position of APY29, located at the interface between the hinge region and the solvent-exposed area. The 4-methylpiperazin-1-yl substituent at the 6-pyrimidine position points toward the solvent-exposed area. With the binding mode information, we propose that the 4-piperazinyl group at the 6-pyrimidine core and the phenoxy group at the 2-pyrimidine core permit further structural modifications by functionalization of the piperazinyl amine and the phenyl substituent, respectively.

**Figure 3:**
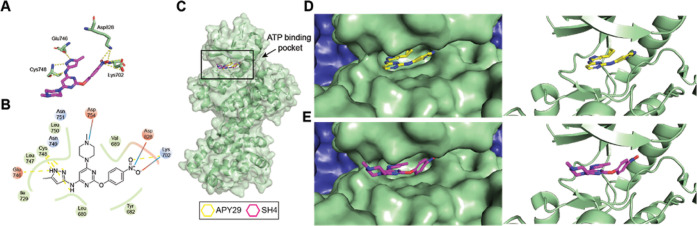
Potential binding mode of SH4 with IRE1α. (A) Docking of SH4 at the APY29-binding site at the APY29–IRE1α complex (PDB 3fbv). Selected residues predicted to interact with SH4 via hydrogen-bond interactions are shown in green backbones; SH4 is in magenta backbones. The hydrogeon bond interactions are indicated by using yellow dotted lines. (B) 2D interaction diagram of SH4 with IRE1. The depicted molecular interactions include hydrophobic interaction (green), ionic interactions (red/blue), hydrogen-bond interactions (yellow dotted lines), and salt bridges (red/blue lines). (C) Overall structure of the IRE1α kinase and RNase domains showing the ATP-binding pocket of the IRE1 monomer, APY29 (shown in yellow backbones) and SH4 (shown in magenta backbones) overlapped at the rectangle-indicated position at the kinase domain (the zoomed-in versions are shown in Panels D and E). (D) APY29 binds at the hinge region of the ATP-binding pocket of the IRE1α kinase domain, shown in surface (left) and cartoon (right) (PDB 3fbv). (E) The docked binding mode between SH4 and IRE1α, mimicking that of APY29, showed the potential modification positions at the 2- and 6-pyrimidine core exposed to the solvent fronts, surface show (left) and cartoon show (right) (green: IRE1 monomer; blue: residual IRE1).

Based on the structure of SH4 and the two positions amenable to further structural modification, a series of the structural analogues with modified R^1^ and R^2^ groups, corresponding to the 4-phenoxy group at the 2-pyrimidine position and the 4-piperazinyl group at the 6-pyrimidine position, were synthesized (Schemes 1 and S1). The choice of the *R* ^1^ substituents (amine, carbonyl, amide, methylsulfonyl, sulfonamide) was focused on groups that can potentially function as hydrogen-bond donors or acceptors to form the key interactions with Asp828 and Lys702.

**Scheme 1:**
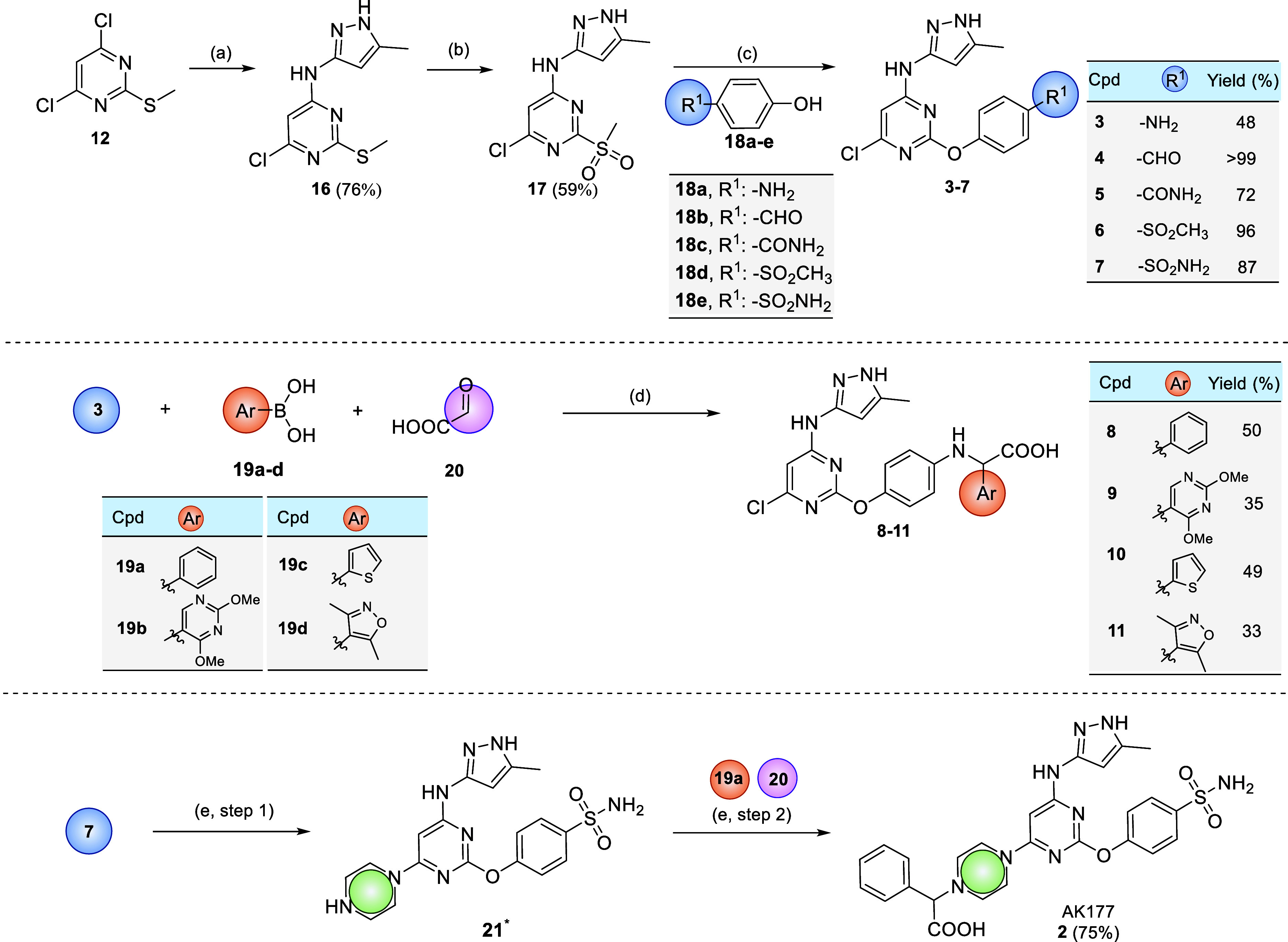
Synthesis of the Functionalized Pyrazolylpyrimidinamines via Petasis Reactions

Reagents and conditions: (a) 5-methyl-1*H*-pyrazol-3-amine, DIPEA, 50 °C, 16 h; (b) 2KHSO_5_. KHSO_4_. K_2_SO_4_, MeOH, 0 °C 30 min, 0 °C-rt, 2 h; (c) KOtBu, *tert*-butanol, 50 °C;^32^ * (c) K_2_CO_3_, DMF, 100 °C, 2 h; (d) HFIP, 3 Å MS, rt, 3–4 h; (e, step 1) piperazine, 1, 4-dioxane, 100 °C, 1 h (*compound 21 was obtained as a crude product and used in the step 2 directly without further purification); (e, step 2) HFIP, 3 Å MS, rt, 3 h.

After testing the activating potency toward both unphosphorylated and phosphorylated IRE1α (Table S1), compound AK177, with the sulfonamide as *R* ^1^ and the installed 2-phenyl-2-(piperazin-1-yl)­acetic acid via PR as *R* ^2^, was found to be the most active compound among the synthesized collection. AK177 showed EC_50_ of 480 nM and 180 nM toward unphosphorylated and phosphorylated IRE1α, respectively, in the FRET assay (Figure S4), which showed a ∼3-fold improvement in comparison with that of SH4 in both cases. For the activating potency toward the unphosphorylated IRE1α, among the pyrazolylpyrimidinamines 3–7 with varying substituents at the *R* ^1^ group on the phenyl moiety, the terminal amine-containing tolyloxy 3 and carbamoyl 5 showed ∼20-fold decrease of activating potency in comparison with that of AK177. Formyl 4, methylsulfonyl 6, and sulfamoyl 7 showed equivalent potency with either SH4 or AK177. Among the petasis products 8–11, 2,4-dimethoxypyrimidinyl 9 exhibited an ∼35-fold decrease of activating potency, indicating a bulky group was not tolerated at this position. Less bulky groups, including the phenyl group of 8 and thiophenyl group of 10, were better tolerated. For the activating potency toward the phosphorylated IRE1α, except for the sulfamoyl compound 7, which showed a 2-fold decrease, all other compounds showed comparative potencies that were up to ∼30-fold decreased in comparison with that of AK177. Collectively, the structure–activity relationship demonstrated that the activating potency toward unphosphorylated IRE1α, instead of the phosphorylated IRE1α, was more prone to be impacted by the performed structural modifications. For the most active compound, AK177, it concentration-dependently cleaved the *XBP1* mRNA probe in the gel-based cleavage assay (Figure S5). It is noteworthy that the choice of *R* ^1^ is only represented by a single group derived from the phenyl boronic acid 19a via the Petasis reaction due to synthetic accessibility, which made it difficult to obtain a complete structure–activity relationship surrounding the pyrazolylpyrimidinamines as IRE1 activators.

The most potent activator AK177 stabilized both the phosphorylated and nonphosphorylated IRE1α upon binding measured by DSF assay in a concentration-dependent manner (Figure 4A). After confirming the RNase-activating potency of AK177 toward IRE1α RNase, we proceeded to evaluate the mechanism of RNase activation by first testing its binding activity toward the kinase domain of IRE1α (Figure 4B) given that binding at the kinase domain is a reported mechanism for a few existing IRE1α RNase activators,^19,21^ also because the pyrimidinamine is a common motif of reported small-molecule kinase inhibitors.^27–29^ Indeed, both SH4 and AK177 showed kinase domain binding affinity with EC_50_ of 127 nM and 188 μM, respectively, tested in the LanthaScreenTM Eu kinase binding assay (Figure 4C). Combining the data from the kinase binding and RNase cleavage assay suggested that the pyrazolylpyrimidinamines are kinase-domain-binding but RNase domain-activating molecules. The potential mechanism is likely via the stabilization of the active conformation of the kinase domain upon binding and inhibition, while in parallel, it is via allosteric activation of the RNase domain.

**Figure 4:**
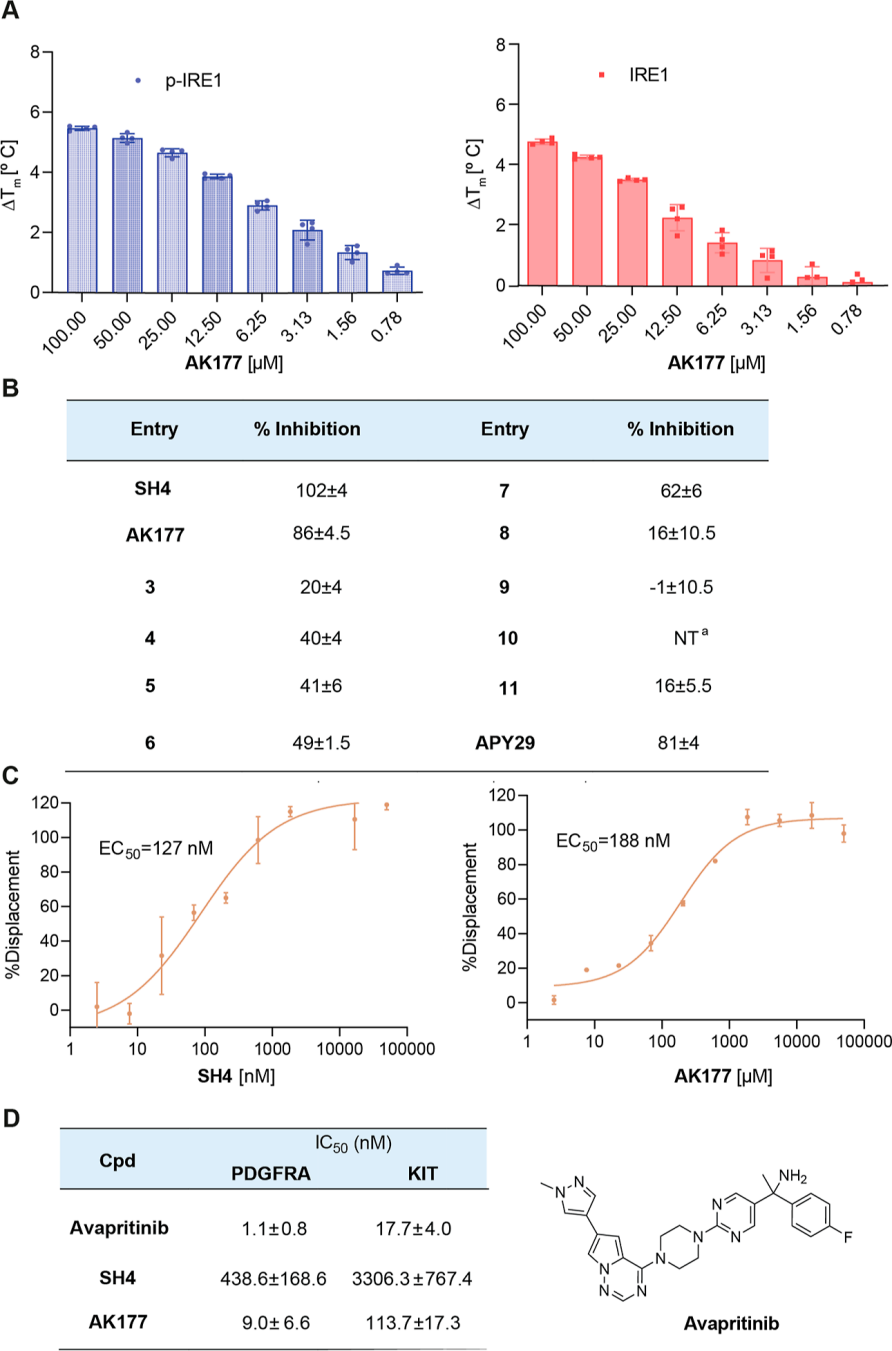
(A) Compound AK177 stabilized IRE1α concentration-dependently in the DSF assay. (B) Binding of selected pyrazolylpyrimidinamines and APY29, a reported IRE1 activator, toward the IRE1α kinase domain. Tested in 5 μM for all compounds. ^a^NT: not tested. (C) Compounds SH4 and AK177 showed comparable binding affinity toward the IRE1α kinase domain in the LanthanScreen Eu kinase assay. (D) Compounds SH4 and AK177 showed different inhibitory activities against PDGFRA and KIT in the homogeneous time-resolved fluorescence (HTRF) assay. The FDA-approved kinase inhibitor avapritinib was used for comparison.

Given the kinase domain binding nature of the pyrimidinamines and to gain insight into the selectivity among kinases, we proceeded to test the representative compounds SH4 and AK177 for their inhibitory activities against two tyrosine kinases (PDGFRA and KIT) that were being evaluated in a separate project targeting gastrointestinal stromal tumors.^30^ Homogeneous time-resolved fluorescence (HTRF) assay results were obtained using the FDA-approved kinase inhibitor avapritinib as a comparison,^31^ which has a piperazinylpyrimidine scaffold analogous to that of SH4 and AK177. As summarized in Figure 4D, SH4 showed ∼400-fold and ∼200-fold reduced inhibitory activities against PDGFRA and KIT, respectively. While AK177 showed only ∼8-fold and ∼6-fold reduced inhibitory activities against PDGFRA and KIT, respectively, it retained potent nanomolar inhibitory activities against both targets. The inhibition results against PDGFRA and KIT indicated that SH4 and AK177 would likely inhibit other kinases across the kinome with varying potency.

Furthermore, we evaluated the selectivity of SH4 and AK177 in a kinase profiling assay against 85 kinases (Figure 5A and B), which echoed the PDGFRA and KIT testing results. Overall, SH4 and AK177 showed varied inhibitory activities against a wide range of kinase targets, with more than 50% inhibition against 37 kinases for SH4 at 5 μM and more than 50% inhibition against 49 kinases for AK177 at 5 μM (Table S2).

**Figure 5:**
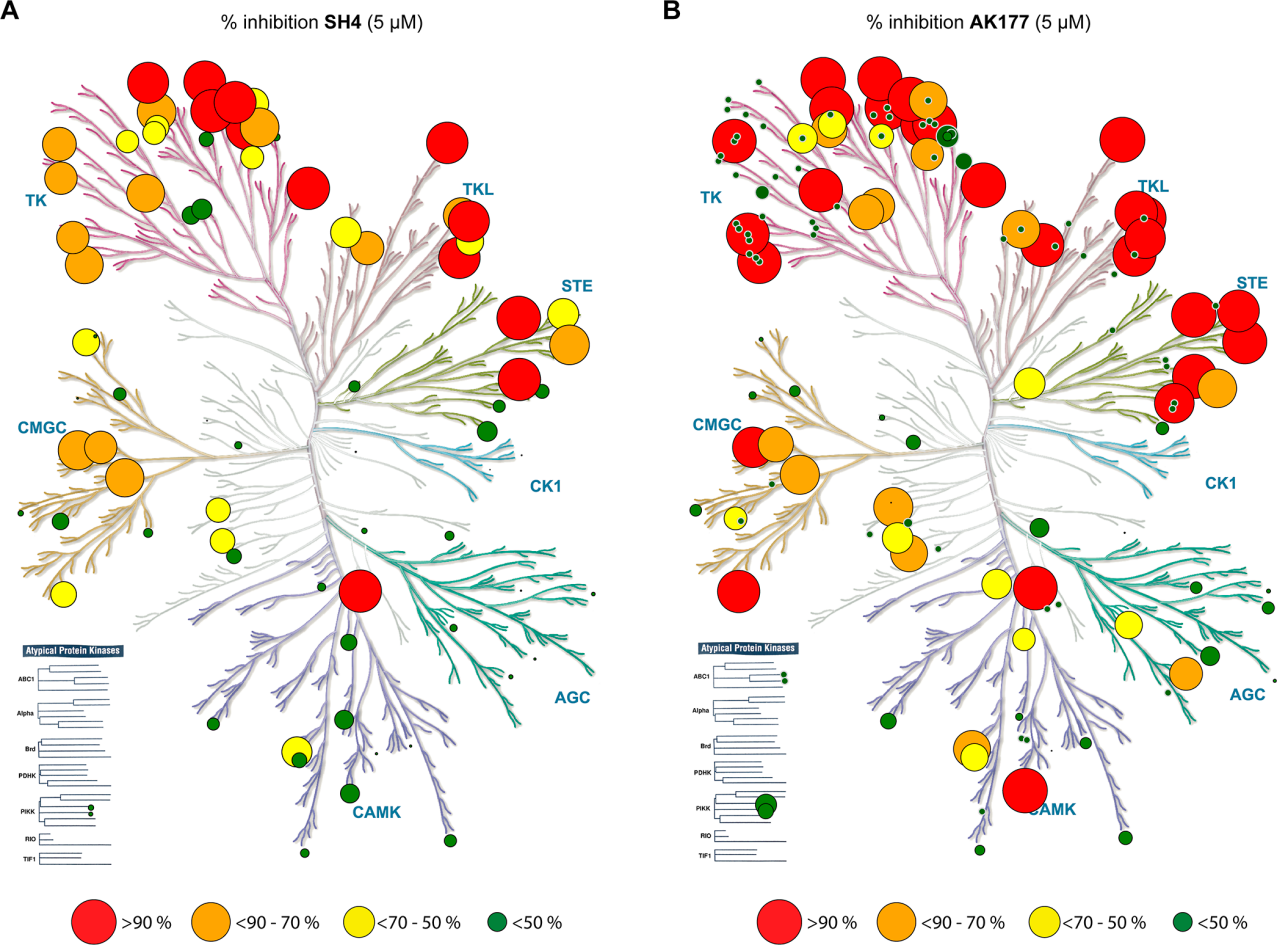
Kinome tree showing the result of kinase profiling for compounds SH4 (A) and AK177 (B) against 85 kinases.

To evaluate the cellular performance of AK177, we measured the impact on *XBP1* mRNA splicing in the MCF-7 cells via reverse transcription-quantitative polymerase chain reaction (RT-qPCR). The results showed that treatment of SH4 led to a concentration-dependent increase of the *XBP1* mRNA splicing (Figure 6A). In comparison, AK177 exhibited less potent activation potency in inducing the *XBP1* mRNA splicing level in MCF-7 cells (Figure 6B). Given the improved IRE1α RNase-activating potency of AK177 in comparison with that of SH4, the reduced activity in inducing the *XBP1* mRNA splicing level observed in the RT-qPCR could be explained by the unfavorable permeability of AK177 due to the presence of the carboxylic acid group. Additionally, the RIDD-mediated degradation of the *Bloc1s1* mRNA was assessed in RT-qPCR. *Bloc1s1* mRNA is expected to be degraded upon RIDD activation. As shown in Figure 6C, SH4 led to a concentration-dependent decrease of the *Bloc1s1* mRNA level in RT-qPCR, with approximately 50% reduction at 20 μM in comparison with that of the control. In comparison, no significant changes were observed in the case of AK177 (Figure 6D), resembling the RT-qPCR results of *XBP1* splicing.

**Figure 6:**
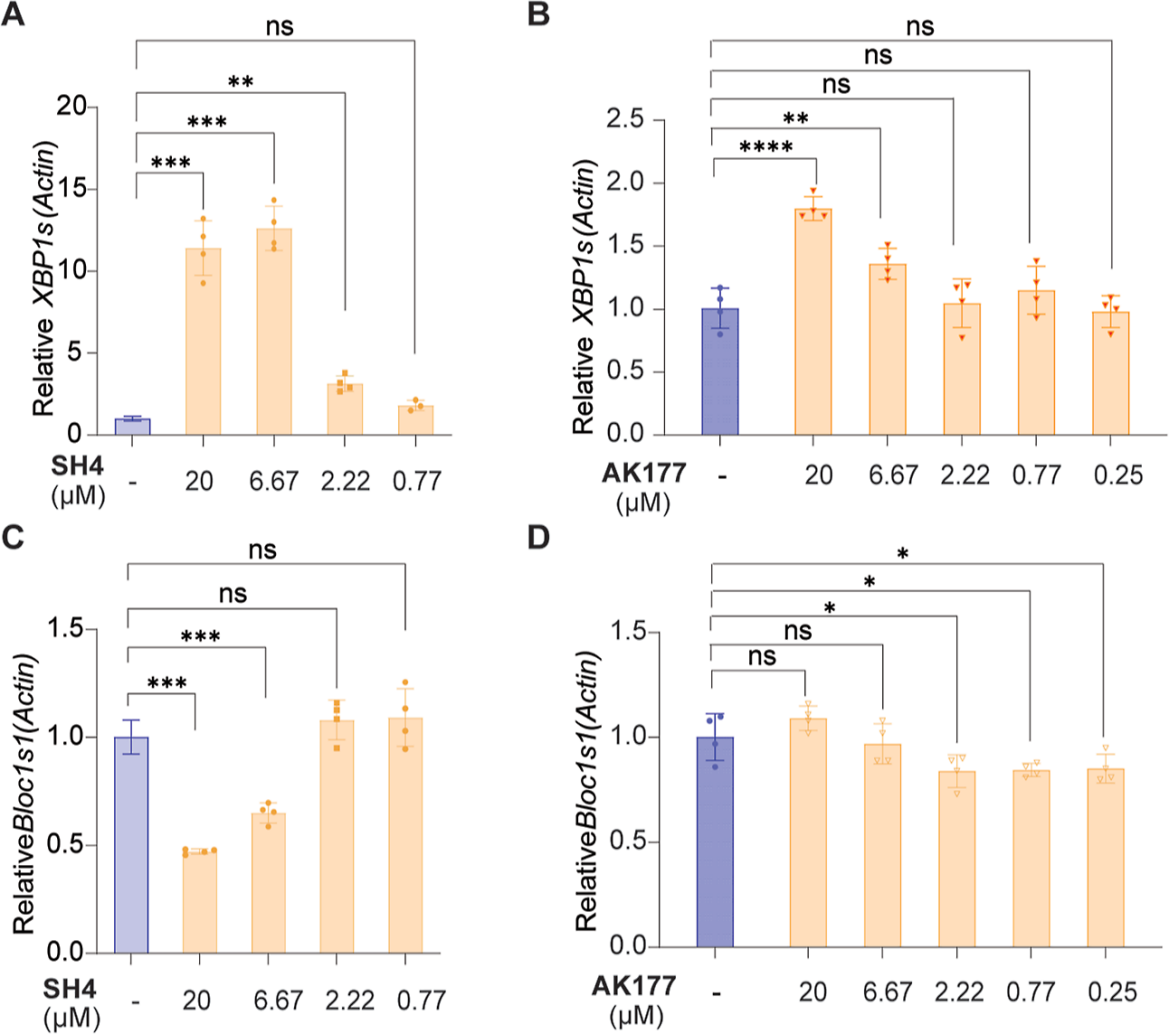
RT-qPCR evaluation of the mRNA levels in MCF-7 cells. (A) SH4 concentration-dependently increased the splicing level of the *XBP1* mRNA. (B) AK177 concentration-dependently increased the splicing level of the *XBP1* mRNA. (C) SH4 concentration-dependently reduced the decay of *Bloc1s1* mRNA via RIDD. (D) AK177 did not show a significant impact on the decay of *Bloc1s1* mRNA via RIDD.

We followed with the protein level evaluation in Western blot (Figure S7). SH4 treatment did lead to the observation of elevated IRE1α protein level, even with the presence of the reported IRE1α inhibitor 4 μ8c.^33^ However, the observed increase in the XBP 1s mRNA level upon SH4 treatment did not lead to a detectable increase in the corresponding XBP 1s protein level; instead, we observed the increased level of unspliced XBP1u protein. The result indicated that the exact mechanism following IRE1α activation and the following downstream network leading to XBP1 splicing remains to be elucidated.

To provide further insights into the cellular activity of SH4 and AK177, we evaluated the cell viability in the cell counting kit-8 assay (CCK-8) in HT-29, MCF-7, and MDA-MB-231 cells. SH4 showed potent inhibitory activity in reducing the cell viability across all tested cell lines, with IC_50_ of 4.9 μM for HT-29, 3.6 μM for MDA-MB-231, and 3.5 μM for MCF-7 cells (Figure 7A). Furthermore, SH4 showed equivalent potency in reducing the cell viability of HEK-293 cells (Figure S6). This reduction in viability of the tested cancer cells can likely be explained by either the hyperactivation of IRE1-mediated pathways or the cytotoxicity and off-target activities of AK177, as reflected by the kinase testing results against PDGFRA and KIT or its metabolites. The exact explanation would therefore warrant further investigation. In contrast, AK177 did not lead to a significant reduction in cell viability (Figure 7B), which aligned with the previously observed weak activation potency in the RT-qPCR evaluation.

**Figure 7:**
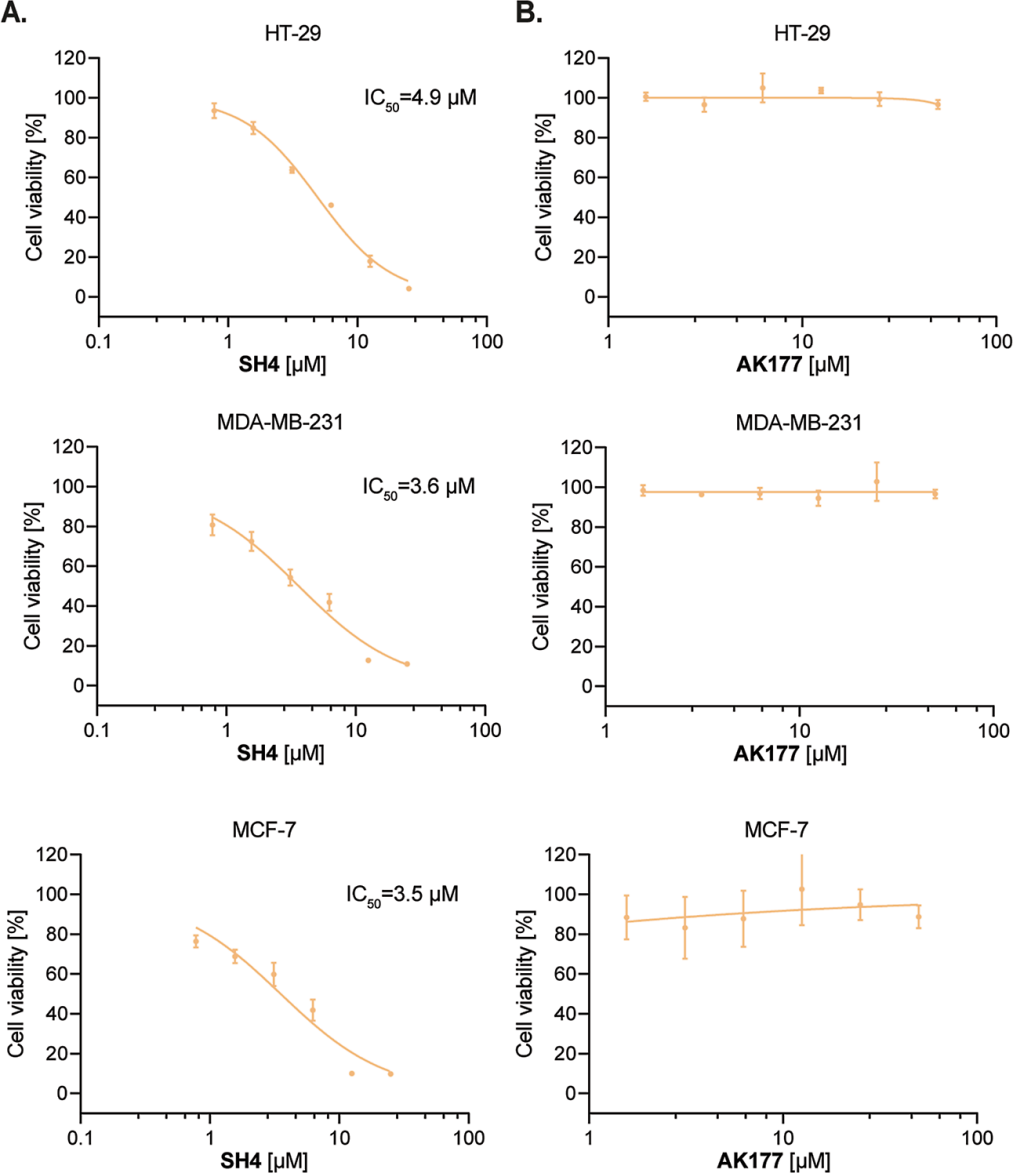
Cell viability of SH4 and AK177 in CCK-8 assay. (A) SH4 concentration-dependently inhibited the proliferation of cancer cells HT29, MCF-7, and MDA-MB-231 in CCK-8. (B) AK177 showed no obvious inhibition against the proliferation of cancer cells HT-29, MCF-7, and MDA-MB-231.

## Conclusion

In this study, we reported the identification of pyrazolylpyrimidinamines as a new series of small-molecule activators of IRE1α RNase activities. The pyrazolylpyrimidinamine SH4, initially identified via a FRET assay, showed single-digit micromolar activation potency toward unphosphorylated IRE1α and submicromolar activation potency toward phosphorylated IRE1α and promoted the IRE1α dimerization measured in mass photometry. A subsequent docking analysis based on a resolved small-molecule–IRE1α complex structure indicated the possibility to accommodate structural modifications at the 2- and 6-pyrimidine core scaffold of SH4 to obtain the most potent activators. Based on this rationale, the following structural modifications enabled by the PR functionalizing the substituents at the 4-piperazinyl group at the 6-pyrimidine core and the phenoxy group at the 2-pyrimidine core were performed. Among the obtained Petasis-reaction-decorated functionalized pyrazolylpyrimidinamines, AK177 showed improved RNase activity with nanomolar activating potency toward both phosphorylated and unphosphorylated IRE1α in the FRET and concentration-dependently stabilized IRE1α upon binding. Both AK177 and SH4 showed equivalent inhibitory activity toward the IRE1α kinase domain, indicating the allosteric RNase activating mechanism via binding at the kinase domain. A partial kinome profiling screening against 85 kinases showed that SH4 and AK177 inhibited a wide range of kinase targets with varied activities, together with the observation that structural analogues of APY29 have been shown to exhibit improved selectivity,^34^ indicating that further structural modifications need to be performed to improve the selectivity for the pyrazolylpyrimidinamines.

Despite the improved activities of AK177 observed in the biochemical assay, the evaluation results in the cellular setting revealed a different story. Although treatment of SH4 potently increased *XBP1* mRNA splicing in MCF-7 cells (>10-fold at the highest tested concentration of 20 μM), the induced *XBP1* mRNA splicing was much less when treating AK177 (<2-fold at the highest tested concentration of 20 μM). Similarly, SH4 led to significantly induced degradation of the *Bloc1s1* mRNA, while AK177 did not show a detectable change in the *Bloc1s1* mRNA level. Furthermore, the potent antiproliferation activities of SH4 against the HT-29, MCF-7, and MDA-MB-231 cells were not echoed by the antiproliferation activities of AK177. Thus, the disparity for AK177 observed between the biochemical and cellular assays is a clear limitation of the study. We propose that this can be explained by the presence of the carboxylic group of AK177, which would negatively impact the permeability of AK177 in cellular assays.

Overall, we present here a series of pyrazolylpyrimidinamines as new small-molecule activators of IRE1α RNase activity. We also demonstrated that further structural decoration realized by the Petasis reaction could lead to compounds with improved RNase activating potency in a biochemical assay, which served as the first examples of applying this type of multicomponent reaction to access bioactive compounds targeting protein–RNA interactions and RNA-cleaving proteins.

## Experimental Section

### Protein Expression and Purification

IRE1α protein was expressed and purified using the Bac-to-Bac system with a reported protocol^35^ and modified in-house. Briefly, the pLIB plasmid encoded IRE1α protein (residues 547-977) was transformed into DH10EmBacY *E. coli* component cells, and the bacmid was purified from the positive cells. The bacmid was further transfected into Sf9 cells to obtain the baculovirus, and the P3 virus was used for protein expression. Sf9 cells were cultured with baculovirus at 27 °C with 120 rpm shaking for 72 h to induce protein expression. The cells were harvested by centrifugation at 4 °C and 4000 rpm for 20 min and resuspended in lysis buffer A containing 50 mM HEPES pH 7.5, 300 mM NaCl, 10% glycerol, 1 mM MgCl_2_, 1 mM TCEP, and 5 mM imidazole. Before cells were lysed, 1 mM PMSF, benzonase (Sigma, E1014), and protease inhibitor cocktail tablet (Sigma, S8830) were supplemented to the cell suspension. After being lysed by sonication, the sample was centrifuged at 4 °C, 20000 rpm for 40 min, and the supernatant was collected and filtered. The filtered sample was purified by a 5 mL nickel-affinity column HisTrap HP (Cytiva) using aforementioned lysis buffer A and lysis buffer B (50 mM HEPES pH 7.5, 300 mM NaCl, 10% glycerol, 1 mM MgCl_2_, 1 mM TCEP, 500 mM imidazole), and the protein was purified with 5% lysis buffer B wash for 10 CV and followed by a gradient of 0–100% lysis buffer B over 20 CV. A ratio of 1:50 TEV protease was added to the protein elution and incubated at 4 °C overnight to cleave the His tag. After his-tag cleavage, the sample was diluted to ∼50 mM NaCl concentration with buffer containing 50 mM HEPES pH 7.5, 1 mM TCEP, and 5% glycerol and loaded onto a 5 mL HiTrap Q HP column (Cytiva). Protein purification was performed with QA buffer (50 mM HEPES pH 7.5, 50 mM NaCl, 1 mM TCEP, 5% glycerol) and QB buffer (50 mM HEPES pH 7.5, 1 M NaCl, 1 mM TCEP, 5% glycerol); the protein was eluted with a gradient from 50 mM to 300 M NaCl over 80 column volumes. Three peaks from the anion exchange column were collected and evaluated through LC–MS. The phosphorylated IRE1 protein was purified from the sample containing three phosphorylations eluted from Q column. The phosphorylated sample was further loaded onto a pre-equilibrated HiLoad 16/600 Superdex 200 pg column (Cytiva) and purified with SEC buffer containing 25 mM HEPES pH 7.5, 250 mM NaCl, 1 mM TCEP, and 10% glycerol. The protein was eluted as a monomeric peak. The dephosphorylated protein was purified from the rest of the proteins from the Q HP column. The proteins were collected and incubated with Lambda protein phosphatase (New England BioLabs, P0753S) to remove the phosphate groups overnight at 4 °C. Then the dephosphorylated protein was further purified using the Superdex 200 pg column (Cytiva) with the buffer mentioned above.

### FRET Assay

The FRET assay was performed using dual-labeled FAM-XBP1-BHQ1 RNA as cleavage substrate (5′FAM-CAUGUCCGCAGCGCAUG-3′BHQ1). The assay was performed in a black 384-well plate (Corning, 4514) with a total reaction volume of 20 μL. The reaction buffer contains 20 mM HEPES, pH 7.5, 50 mM potassium acetate, 1 mM magnesium acetate, 1 mM DTT, and 0.05%(v/v) Triton X-100. IRE1 protein (5 μL) was incubated with compound (5 μL, final 0.5% DMSO) for 30 min at room temperature; then 10 μL of dual labeled RNA substrate was added into each well, and the fluorescence was kinetically measured over 1 h at 25 °C using a TECAN Spark plate reader with 485 nm excitation wavelength and 535 nm emission wavelength. The linear range of the curve was used and analyzed. The final concentration for the dephosphorylated IRE1α FRET assay is 40 nM IRE1α protein with a 100 nM RNA substrate. The final concentration for the phosphorylated IRE1α FRET assay is 4 nM p-IRE1α protein with 200 nM RNA substrate.

### Microscale Thermophoresis (MST)

Following the manufacturer’s protocol, the dephosphorylated and phosphorylated IRE1α proteins were labeled and purified using the Protein Labeling Kit RED-NHS Second Generation (NanoTemper, MO-L011). The binding experiments were conducted according to the instructions of a Monolith Nt.115 instrument (NanoTemper) with an excitation power of 20%. The compounds were serially diluted 2-fold and incubated with the labeled IRE1α protein at a final concentration of 20 nM. The highest compound concentration measured was 250 μM, with the final DMSO concentration kept at 0.5%. Data were analyzed using the manufacturer’s software, and results from three individual measurements were obtained for analysis.

### Cross-Linking Assay

Phosphorylated IREα was incubated with either compound SH4, G7658, or DMSO for 30 min at room temperature, with the final concentration of 2 μM protein and 100 μM compound, with the final 0.5% (v/v) DMSO in the aforementioned reaction buffer. After incubation, DSS (Thermo Fisher, A39267) was added to a final concentration of 250 μM to initiate cross-linking at room temperature for 1 h. Subsequently, a final concentration of 50 mM Tris (pH 7.5) was added to quench the reaction. Mass analysis was performed using a mass photometry instrument (Refeyn Ltd.) according to the manufacturer’s instructions with PBS buffer. Briefly, samples were diluted to a final protein concentration of 100 nM with PBS buffer. To process the measurement, 18 μL of PBS was dropped to the center of a gasket on the glass slide to adjust the focus. Then 2 μL of the diluted sample was added to the PBS drop, gently mixed, and recorded. The data were processed by using Discover MP software. The BSA and TG protein mixture was used for calibration.

### Molecular Docking Analysis

The molecular docking analysis was performed by using Schroedinger Maestro 12.3. The crystal structure of oligomeric IRE1 bound to APY29 was used (PDB: 3FBV) for docking. To prepare the IRE1α conformation, the Protein Preparation Wizard was used to add and optimize the position of hydrogen atoms, create disulfide bonds, and delete water molecules for a chosen monomer. Protonation states were assigned at a pH of 7.0, and hydrogen atoms were minimized using Refine of the Protein Preparation Wizard. The 3D structures and all possible electronic states at a pH of 7.0 ± 2.0 of SH4 were prepared after performing energy minimization by MM2 with PerkinElmer Chem3D 20.1, and the chemical states were generated with the ligand preparation module with the default settings. A 10 Å × 10 Å × 10 Å grid was generated at the ATP binding pocket of IRE1, and all electronic states of the ligand were docked to the grid using the Glide dock module. The docking results evaluation was conducted on the basis of the orientation of SH4, interactions between SH4 and IRE1α, and the docking scores. The visualization of the docking results was performed by using PyMOL 2.4.1.

### DSF Assay

The DSF assay was performed in a white 96-well PRC plate (Starlab, no. E1403-1209) using a Bio-Rad CFX96 Real-Time PCR detection system with a total volume of 20 μL. IRE1 protein, compound, and the SYPRO orange dye (Sigma S5692) was diluted in assay buffer (PBS buffer supplied with 2 mM DTT) with the final protein concentration of 1 μM (for both IRE1α and p-IRE1α protein) and the final dye concentration of 5×. The melting curve was measured using the FRET scan mode with a temperature increase of 1 °C for 30 s at a temperature range from 25 to 95 °C. The data was analyzed using the Boltzmann sigmoidal fit with GraphPad Prism.

### LanthaScreenTM Eu Kinase Binding Assay

LanthaScreenTM Eu kinase binding assays were conducted by SelectScreen Kinase Profiling Services (Thermo Fisher) using Alexa Fluor conjugated kinase tracer, tagged kinase protein, and Eu-labeled antitag antibody. The binding of the tracer to the kinase will form the tracer-kinase complex, resulting in a high FRET signal with the Eulabeled antitag antibody. Displacement of the tracer by a kinase inhibitor led to a decreased intensity of the FRET signal. For the IRE1α (ERN1) kinase binding assay, 5 nM of ERN1, 2 nM of Eu-anti-GST, and 100 nM tracer 236 (with *K*_d_ = 160 nM) were used. For the IRE1β (ERN2) kinase binding assay, 5 nMof ERN2, 2 nMof Eu-anti-GST, and 100 nM tracer 236 (with *K*_d_ = 108 nM) were used. Buffer A containing 50 mM HEPES (pH 7.5), 0.01% BRIJ-35, 10 mM MgCl2, and 1 mM EGTA was used.

### HTRF Assay

All supplies for the KIT and PDGFRA HTRF assay kit were purchased from CisBio (Bagnolssur, Cez̀e, France). Active enzymes were purchased from ProQinase [KIT-wt [0997-0000-1 (010)] and PDGFRA-wt (#1057-0000-1)]. Small-volume (25 μL fill volume) white round-bottom 384-well plates were obtained from Greiner Bio-one GmbH (Solingen, Germany).

### Activity-Based Assay

The biochemical half-maximal inhibitory concentrations (IC_50_) were determined with the TK HTRF KinEASE assay (Cisbio) according to the manufacturer’s instructions. Briefly, 5 μL of kinase solution and 2.5 μL of inhibitor solution (8% DMSO in HTRF buffer) were incubated for 30 min before the reaction was started by the addition of 2.5 μL of starting solution containing ATP and substrate peptide. ATP concentrations were set at their respective KM values (200 μM for KIT-wt and 57 μM for PDGFRA-wt). The following substrate concentrations were used: 550 nM for KIT-wt and 455 μM for PDGFRA-wt. After reaction completion (KIT-wt: 20 min; PDGFRA-wt: 40 min), 10 μL of the stop solution was added. The FRET signal was measured with an EnVision plate reader (PerkinElmer, Waltham, MA, US) (λex 620 nm/λem 665 nm). The quotient of both intensities was recorded at 8 different inhibitor concentrations, and data were fit to a Hill 4-parameter equation with the Quattro software suite (Quattro Research GmbH, Martinsried, Germany). Each reaction was performed in duplicate, and at least three independent determinations of each IC_50_ were made.

### Kinase Profiling

Kinase profiling was performed by the FreeChoice Kinase Screening Service provided by ReactionBiology with a 33P PanQinase Assay for compounds SH4 and AK177 (both at 5 μM) against 85 different kinases. The respective activities tested for the 85 kinases are listed in Table S2 (the kinome trees shown in Figure 5 were generated using the KinMap web portal).

### Cell Culture

The MDA-MB-231, MCF-7, and HT-29 cells were purchased from DSMZ (German Collection of Microorganisms and Cell Cultures). The HEK293 cells were purchased from ATCC (American Type Culture Collection). All cell lines were cultured in high-glucose DMEM medium (Gibco 61965026) with added 10% FBS (Gibco 10500064) and 1% penicillin–streptomycin (Gibco 15140122) at 37 °C at 5% CO_2_.

### RT-qPCR

The MCF-7 cells were seeded in 6-well plates with 1.000.000 cells per well. Cells were precultured overnight and treated with indicated compound concentrations (0.1% DMSO) or Control (0.1% DMSO) for 24 h. RNA was purified using an RNeasy Mini Kit (Qiagen, 74104), and 500 ng of RNA was transcribed to cDNA using the High-Capacity cDNA Reverse Transcription Kit (Applied Biosystems, 4368814). The qPCR was performed using PowerUp SYBR Green Master Mix (Thermo Fisher, A25742) with 10 μL of volumina. Cycling was performed using Bio-Rad CFX96 Real-Time PCR Detection System following standard cycling mode (primer Tm ≥ 60 °C). The following list of primers were used:

Actin forwardGCGAGAAGATGACCCAGATCActin
reverseCCAGTGGTACGGCCAGAGGXBP 1s forwardGAGTCCGCAGCAGGTGXBP 1s reversedCAATACCGCCAGAATCCABloc1s1 forwardGGACCTGTAGGGTCTTCACCTBloc1s1 reverseAGGAGGCGAGAGGCTATCAC

### Cell Viability Assay

MCF-7, HT-29, or MDA-MB-231 cells were seeded in 96-well plates, with 5000 cells per well for MDA-MB-231 and HT-29 and 10000 cells per well for MCF-7. Cells were precultured overnight and afterward treated with indicated concentrations of compound (0.5% DMSO) or Control (0.5% DMSO) for 72 h. CCK-8 (Vazyme, A311) was added to the wells and incubated for 2h at 37 °C, and the absorbance was measured at 450 nM using a TECAN Spark plate reader. Cell viability was calculated using $100\%-100\frac{control-X}{control-blank}$ . Control: absorbance of 0.5% DMSO; X: absorbance of compound treatment; blank: absorbance of medium only.

### Western Blot

HT-29 cells were seeded into 6-well plates with 1 × 10^6^ cells per well and precultured overnight. After attachment, cells were treated with indicated compound concentrations and a final concentration of 0.5% DMSO for indicated times. Treated cells were washed 2 times with ice-cold PBS and lysed by RIPA buffer supplemented with 1× protease inhibitor cocktail (Sigma, P8340) for 30 min, continued by removal of cell debris via centrifugation for 10 min at 18,000 rpm. The protein concentration was measured using a BCA protein assay kit (Thermo Scientific, 23227), and 30 μg of protein was mixed with 1 x LDS sample buffer (Invitrogen, NP0007) and denatured at 70 °C for 10 min. The protein solutions were loaded onto a NuPage 4–12% gel (Invitrogen, NP0329) and separated in MES-SDS-running buffer (Invitrogen, B0002). Proteins were transferred onto a 0.45 μM PVDF membrane (Bio-Rad, 1704274) using the Trans-Blot Turbo Transfer System with the preprogrammed standard SD protocol (up to 1.0 A; 25 V; 30 min). Membranes were blocked in TBST buffer with 5% skim-milk (Roth, T145.2), washed with TBST, and incubated with primary antibody IRE1α (Cell Signaling Technology, #3294 s, 1:1000) and XBP 1s (Cell Signaling Technology, #12782 s, 1:1000) overnight at 4 °C with shaking. Afterward, membranes were washed with TBST and incubated with Goat Anti-Rabbit (Proteintech, SA00001-2, 1:10000) at RT for 1 h. After washing with TBST, membranes were visualized using an Amersham ECL prime western blotting detection kit (Cytiva, RPN2232) and imaged with a ChemiDoc MP imaging system (Bio-Rad). Membrane treated with XBP 1s antibody was stripped using Restore PLUS Western Blot Stripping-Puffer (Thermo Scientific, 46430), washed with TBST, and incubated with GAPDH (Proteintech, 10494-1-AP, 1:10000). The membrane was washed, treated with Goat Anti-Rabbit, and imaged as described before.

## Supplementary Material



## Notes

^1^H NMR, ^13^C NMR, and UPLC-MS spectra (PDF)

## Abbreviations

BHQ: black hole quencher
CCK-8: cell counting kit-8 assay
FRET: Förster resonance energy transfer
IRE1α: inositol-requiring enzyme 1 alpha;
MST: microscale thermophoresis
PR: Petasis boron-Mannich reaction
RIDD: regulated IRE1-dependent decay
RNase: endoribonuclease
UPR: unfolded protein response
XBP1: X-box binding protein 1.
