# Supplementary material for: Pyrazolylpyrimidinamines Decorated via Petasis Reaction as Small-Molecule Activators of the RNA-Degrading Ribonuclease IRE1α
Source: ACS Bio Med Chem Au. 2026 Feb 27;6(2):118–29. doi: 10.1021/acsbiomedchemau.5c00161 (PMC13087800; doi:10.1021/acsbiomedchemau.5c00161)
Supplement: Supplementary file 1 [file bg5c00161_si_001.pdf]

Supporting Information

Pyrazolylpyrimidinamines Decorated via Petasis Reaction as Small-Molecule Activators of the RNA-Degrading Ribonuclease IRE1α

Amrutha K. Avathan Veettil,[1-3,#] Yang Liu,[1-3,#] Leon Wagner,[1-3,#] Oguz Hastürk,[1-3] Nguyen Song Thu Huynh,[1-3] Giorgia Mancino,[3,4] Maria Beerbaum,[3,4] and Peng Wu[1-3*]

[1]Chemical Genomics Centre, Max Planck Institute of Molecular Physiology, Dortmund 44227, Germany

[2]Department of Chemical Biology, Max Planck Institute of Molecular Physiology, Dortmund 44227, Germany

[3]Department of Chemistry and Chemical Biology, TU Dortmund University, Dortmund 44227, Germany

[4]Drug Discovery Hub Dortmund (DDHD), Zentrum für Integrierte Wirkstoffforschung (ZIW), Dortmund 44227, Germany

TU Dortmund University, Dortmund 44227, Germany

*Correspondence: P. Wu, Email: peng.wu@mpi-dortmund.mpg.de; peng.wu@tu-dortmund.de

Table of contents

Supplementary Figures.. S03

Supplementary Tables.. S10

General Chemistry Information.. S14

Supplementary Scheme.. S15

Synthetic Procedures and Characterization data.. S16

LC-MS Spectra... S23

NMR Spectra... S36

References.. S46

Supplementary Figures

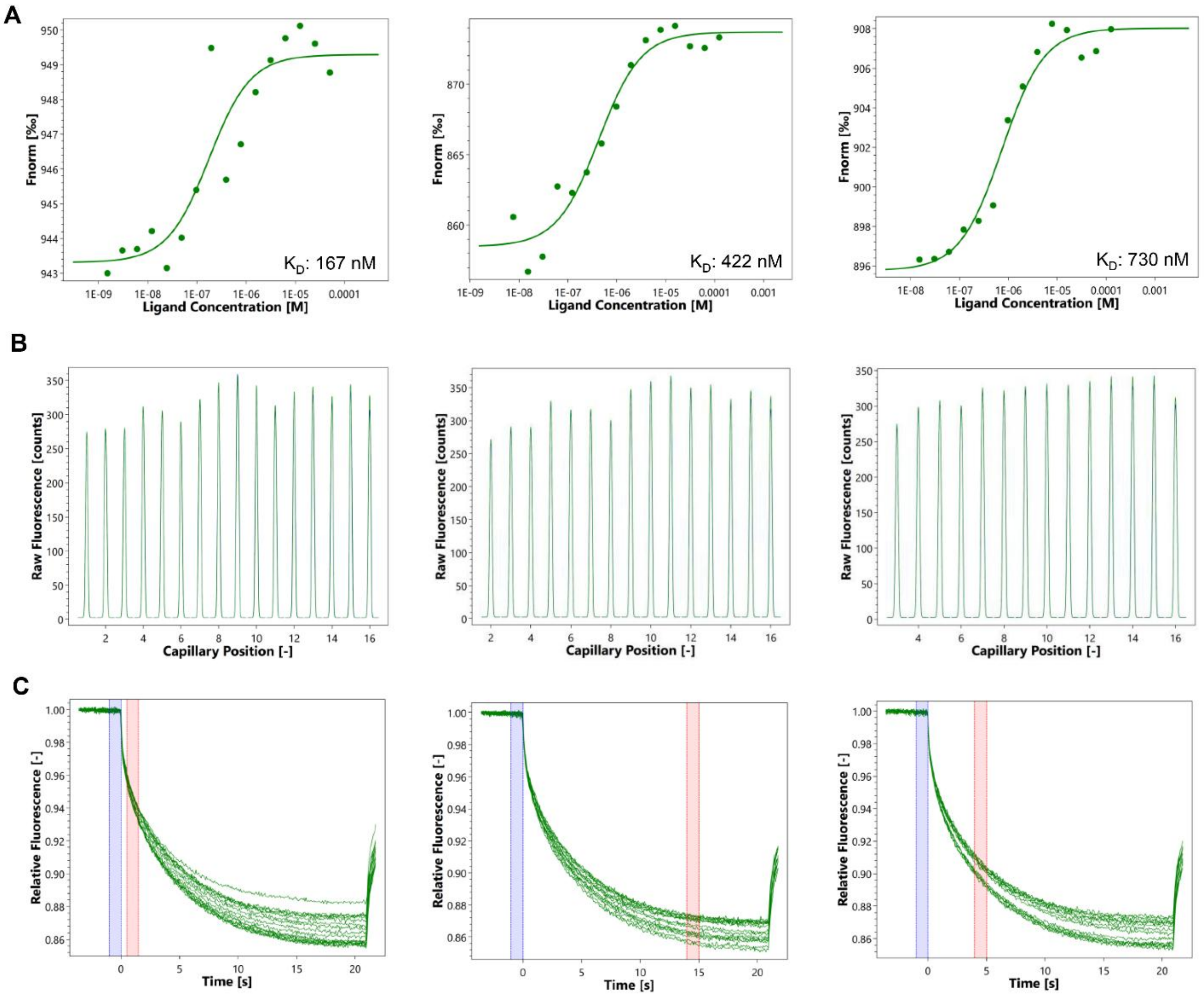

Figure S1. Measurement of the binding affinity in microscale thermophoresis (MST). (A) Dose response curves that showed the binding affinity ranging between 167 nM and 730 nM for SH4 towards IRE1α in MST, performed in triplicates. (B) Capillary scans. (C) MST traces.

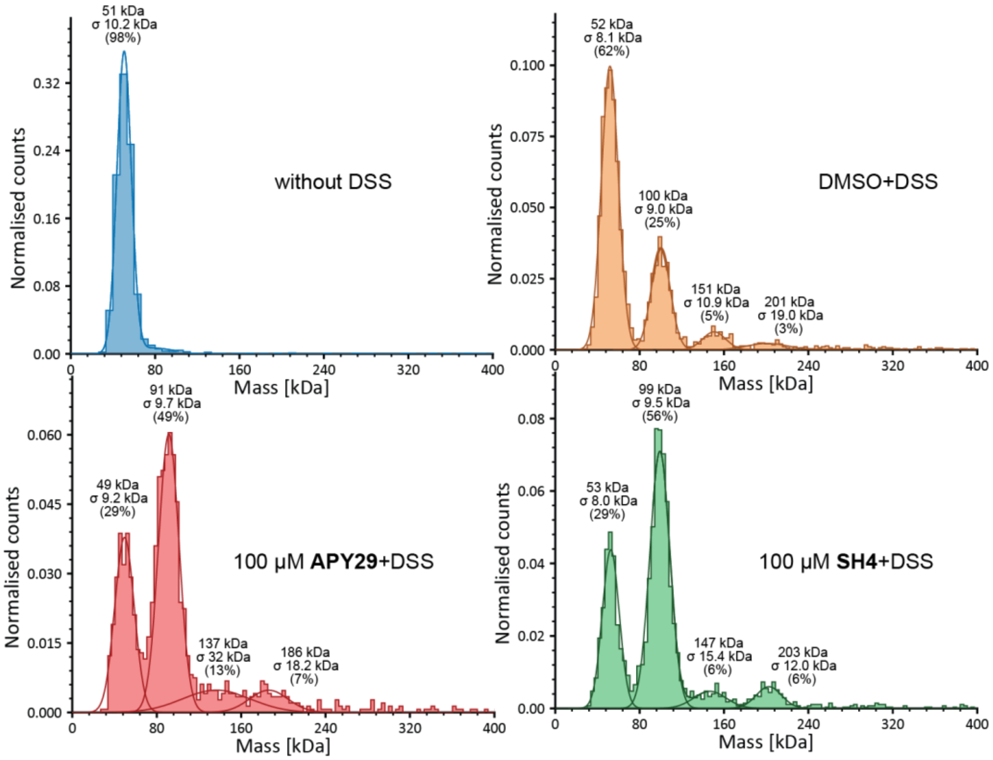

Figure S2. SH4 activates IRE1α activity by promoting the dimerization, measured in the Refeyn mass photometry (the reported activator APY-29 was used as a comparison), shown here is the result from the biological repeat II (in addition to the repeat I result shown in Figure 2G)

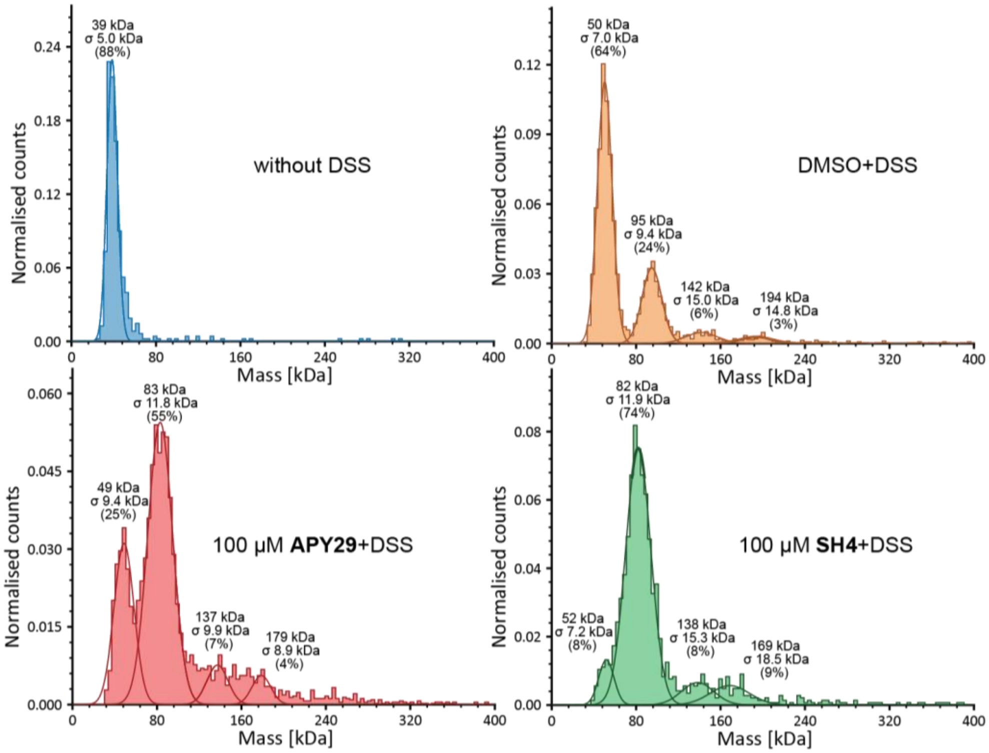

Figure S3. SH4 activates IRE1α activity by promoting the dimerization, measured in the Refeyn mass photometry (the reported activator APY-29 was used as a comparison), shown here is the result from the biological repeat III (in addition to the repeats I and II results shown in Figure 2G and Figure S2, respectively).

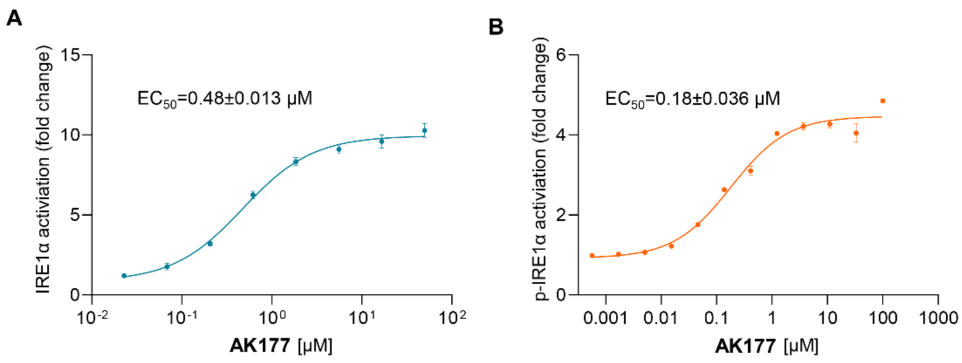

Figure S4. The compound AK177 activated unphosphorylated (A) and phosphorylated IRE1α (B) in the FRET assay, data are presented as mean±SD.

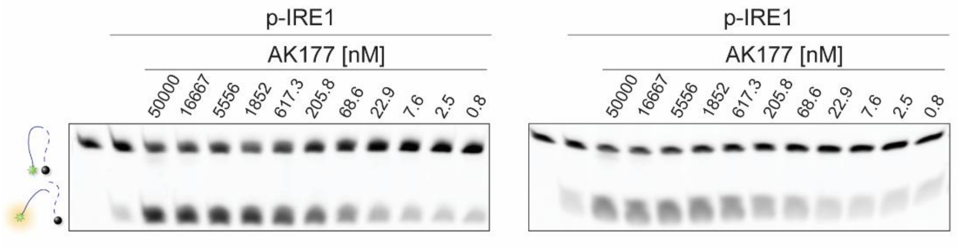

Figure S5. The compound AK177 concentration-dependently activated phosphorylated IRE1α in the gel-based cleavage assay.

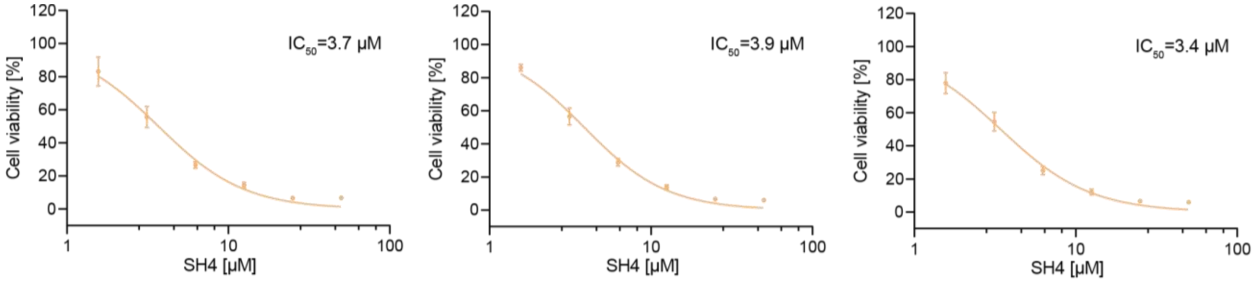

Figure S6. Testing of the cell viability of compound SH4 against HEK-293 cells in the cell counting kit-8 assay (CCK-8).

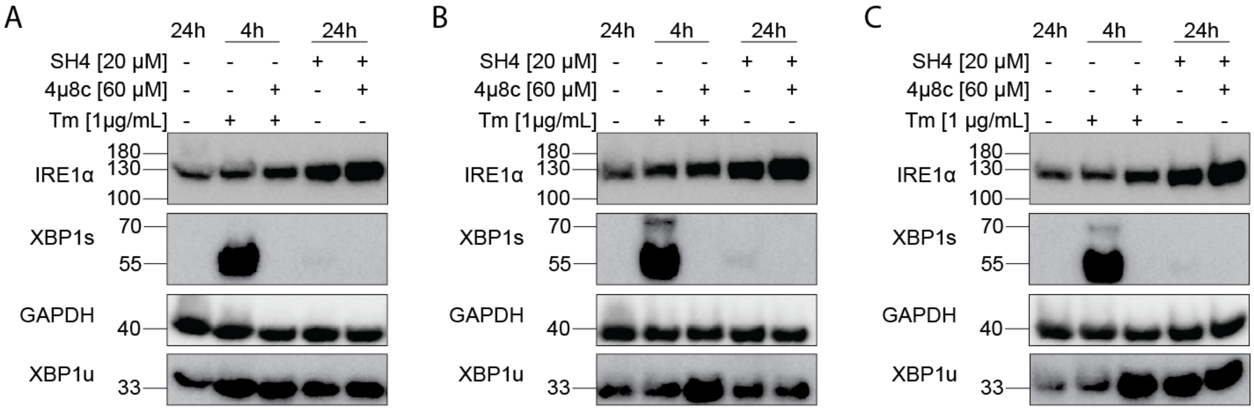

Figure S7. Measurement of IRE1α and XBP1s protein levels in HT-29 cells by Western blot. Use of the XBP1s antibody led to an additional band at 33 kDa, corresponding to unspliced XBP1. SH4 treatment did lead to detectable XBP1s levels. 4µ8c: reported IRE1α inhibitor.Tm: Tunicamycin. Panels **A**-**C** show the three biological replicates.

Table S1. Activation towards unphosphorylated IRE1α and phosphorylated IRE1α (p-IRE1α) measured in the FRET assay for compounds **1** –**11**.

Compound	Structure	IRE1α, EC$_{50}$ (μM)	p-IRE1α, EC$_{50}$ (μM)
SH4 (**1**)	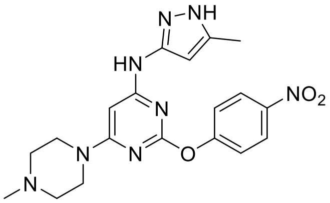	1.31	0.49
AK177 (**2**)	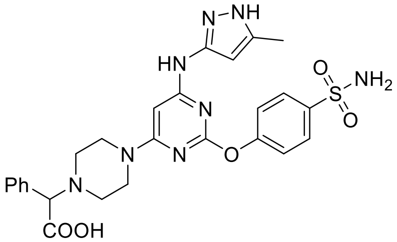	0.48	0.18
**3**	R^1: NH_2	10.02	5.39
**4**	R^1: CHO	0.87	2.52
**5**	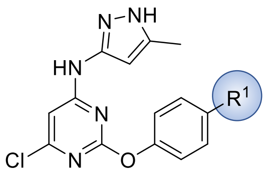 R^1: $CONH_2$	8.35	2.27
**6**	R^1: SO_2CH_3	0.65	1.10
**7**	R^1: SO_2NH_2	1.04	0.45
**8**	Ar: 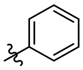	2.71	4.96
**9**	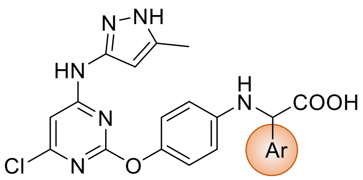 Ar: 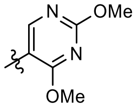	17.66	5.49
**10**	Ar: 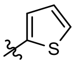	2.23	4.28
**11**	Ar: 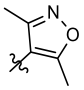	ND[a]	4.23

Table S2. Kinase profiling data of SH4 (5 µM) and AK177 (5 µM), related to Figure 5.

Index	Kinase	Residual activities (% of control)					
		SH4			AK177		
		Dup. 1	Dup. 2	Mean	Dup. 1	Dup. 2	Mean
1	ABL1	3	3	3	16	15	16
2	ACVR1	5	4	4	5	11	8
3	ACVR1B	6	5	5	19	23	21
4	AKT1	95	87	91	95	88	92
5	AMPKalpha1	19	17	18	33	33	33
6	AuroraA	3	3	3	6	6	6
7	BMPR1B	10	10	10	37	40	39
8	BRSK2	44	34	39	71	64	67
9	BTK	8	3	5	29	27	28
10	CAMK1D	74	66	70	76	70	73
11	CAMKK2	32	32	32	45	44	45
12	CDC7/DBF4	72	65	68	88	80	84
13	CDK1/CycB1	6	6	6	44	42	43
14	CDK8/CycC	103	100	102	102	99	100
15	CHK1	37	30	34	85	79	82
16	CK1alpha1	108	94	101	100	95	98
17	CK1gamma2	101	102	101	99	98	99
18	CLK2	90	78	84	106	87	96
19	DAPK3	73	57	65	79	67	73
20	DDR2	2	2	2	15	14	14
21	DNAPK	69	63	66	90	92	91
22	DYRK1A	95	88	91	41	39	40
23	DYRK3	110	91	101	96	91	93
24	EEF2K	132	99	115	118	102	110
25	EGFR	81	78	80	103	90	96
26	EPHA2	2	5	3	24	9	16

27	EPHB4	38	31	34	51	49	50
28	ERBB2	83	73	78	89	77	83
29	ERK2	76	72	74	92	84	88
30	ERK7	15	12	13	15	14	15
31	FAK	22	21	22	69	52	60
32	FGFR1	3	1	2	8	7	7
33	FLT3	1	2	2	4	3	3
34	GSG2	106	101	104	102	91	97
35	GSK3beta	6	14	10	14	12	13
36	HIPK4	76	76	76	74	70	72
37	ICK	22	23	22	27	29	28
38	IGF1R	40	36	38	50	43	47
39	IKKbeta	100	96	98	91	83	87
40	IKKepsilon	14	12	13	47	45	46
41	INSR	23	19	21	51	48	49
42	JAK3	1	1	1	7	6	6
43	JNK1	54	45	49	69	57	63
44	KIT	16	16	16	32	29	31
45	LCK	15	14	14	28	27	28
46	LIMK1	4	4	4	26	25	26
47	MAP4K1	2	1	1	2	3	3
48	MAPKAPK2	113	83	98	105	85	95
49	MARK2	80	77	78	84	79	81
50	MEK1	12	12	12	70	77	73
51	MET	45	47	46	74	62	68
52	MKK6 SDTD	69	75	72	55	57	56
53	MLK3	0	1	0	8	9	9
54	MST1	7	7	7	33	28	31
55	MTOR	54	52	53	89	88	88
56	NDR1	42	36	39	87	80	84
57	NEK1	105	104	105	112	100	106

58	NIK	31	32	31	79	71	75
59	p38alpha	105	102	103	108	100	104
60	PAK4	12	16	14	73	64	69
61	PDGFRbeta	1	3	2	17	11	14
62	PHKG2	69	80	75	95	95	95
63	PIM1	110	104	107	108	101	105
64	PKA	54	63	58	97	92	95
65	PKCbeta1	95	91	93	104	116	110
66	PKCmu	88	81	84	63	53	58
67	PLK1	98	96	97	98	101	100
68	RET	1	2	1	5	5	5
69	RIPK2	16	16	16	34	30	32
70	ROCK1	27	27	27	88	94	91
71	RPS6KA3	1	1	1	58	58	58
72	S6K	78	69	74	92	103	97
73	SGK3	76	71	73	96	117	106
74	SRC	3	2	3	27	23	25
75	STK33	53	48	50	62	65	64
76	SYK	21	21	21	56	55	55
77	TAOK2	1	2	2	6	7	6
78	TGFBR1	2	2	2	9	9	9
79	TIE2	29	27	28	52	46	49
80	TLK1	62	54	58	85	90	88
81	TNIK	2	2	2	15	9	12
82	TRKB	1	2	1	9	9	9
83	TTK	88	89	88	100	97	98
84	ULK2	18	18	18	68	66	67
85	VEGFR2	2	2	2	8	5	7

General Chemistry Information

All reagents and solvents used for the synthesis were purchased from Sigma-Aldrich, Thermo Fischer Scientific, TCI chemicals, Acros, and BLD Pharm. Chromatographic purifications were carried out using a Büchi flash chromatography system using FlashPure Ecoflex silica gel (50μm irregular) cartridge or preparative HPLC systems (Nucleodur C18 gravity column, 5 μm) using appropriate solvent gradients. All solvents used for chromatographic purifications were laboratory grade and purchased from VWR chemicals. Reaction progress was monitored using thin-layer chromatography (TLC) carried out using silica gel-coated aluminum plates (Merck 60 F254). The compounds were visualized under ultraviolet light at wavelengths of 254 nm and/or 356 nm. The purity of the synthesized final compounds was assessed by LC-MS analysis (Agilent 1260 II Infinity system with a mass detector; column used: InfinityLab Poroshell 120 EC-C18, 2.1 × 150, 2.7 μm). NMR spectra were recorded using Bruker DRX400 (400 MHz), DRX500 (500 MHz), DRX600 (600 MHz), and DRX700 (700 MHz) spectrometers in CD_3OD or DMSO-d_6. Reported NMR data include chemical shifts (in ppm), signal patterns (s = singlet, d = doublet, t = triplet, dd = double of doublet, m = multiplet), *J*-coupling constants in Hz, and integration values. High-resolution mass spectrometry was recorded on a compact QTOF (Bruker Daltonics GmbH & Co. KG, Bremen, Germany) coupled to an Agilent 1260 Infinity II system (Agilent Technologies, Waldbronn, Germany) with a G7129A autosampler, a G7116A column oven, a G7117C photodiode array detector and a G7111B quaternary pump system by ESI or APCI ionization methods.

Supplementary Scheme

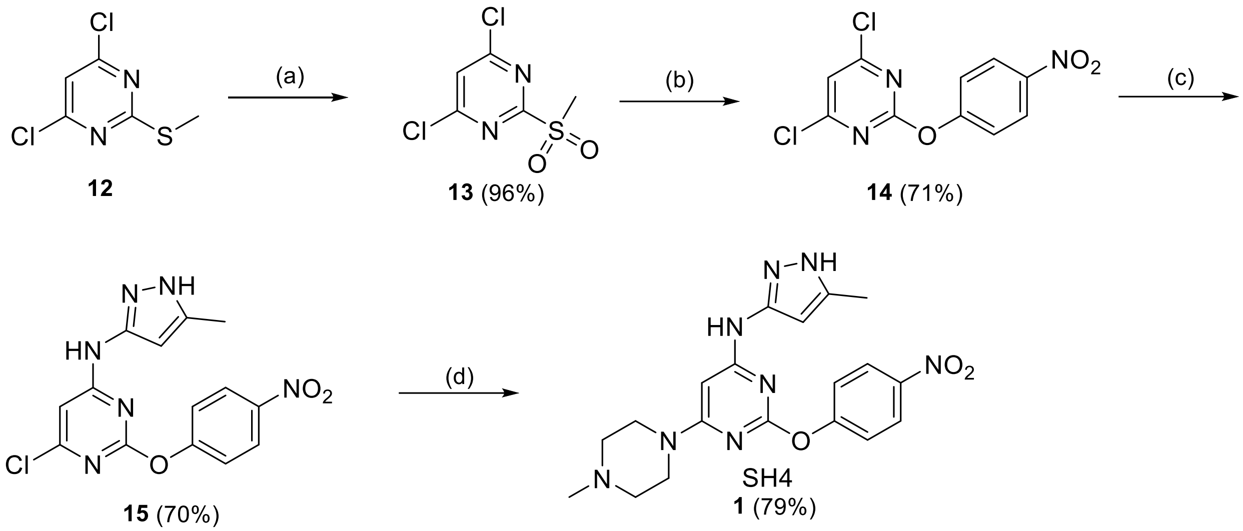

Scheme S1. Synthesis of the pyrazolylpyrimidinamine SH4 (1). Reagents and conditions: (a) *m*CPBA, DCM, 0 °C-rt, 72h; (b) 4-nitrophenol, NaH, DMF, Ar, 0 °C-rt, 4h; (c) 5-methyl-1*H*-pyrazol-3-amine, DIPEA, DMF, 80 °C, 12 h; (d) 1-methylpiperazine, 10 °C, 30 min.

Synthetic Procedures and Characterization Data

6-Chloro-*N*-(5-methyl-1H-pyrazol-3-yl)-2-(methylthio)pyrimidin-4-amine (16)

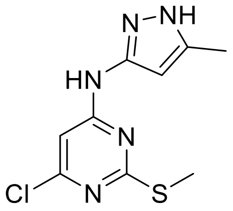

Synthesized by following reported literature procedure and spectroscopic data were consistent with those reported in literature.[1] ^{1}H NMR (500 MHz, DMSO-d_6) δ = 12.12 (s, 1H), 10.19 (s, 1H), 7.45-5.82 (br m, 2H), 2.49 (s, 3H), 2.21 (s, 3H).

6-Chloro-*N*-(5-methyl-1*H*-pyrazol-3-yl)-2-(methylsulfonyl)pyrimidin-4-amine (17)

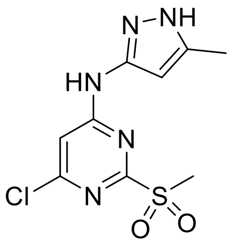

Synthesized by following reported literature procedure and spectroscopic data were consistent with those reported in literature.[1] ^{1}H NMR (500 MHz, DMSO-d_6) δ = 12.29 (s, 1H), 10.94 (s, 1H), 7.98 -5.82 (br m, 2H), 3.35 (s, 3H), 2.23 (s, 3H).

2-(4-Aminophenoxy)-6-chloro-*N*-(5-methyl-1*H*-pyrazol-3-yl)pyrimidin-4-amine (IA180 or 3)

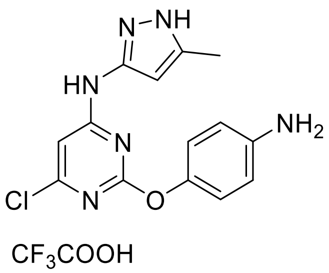

Potassium carbonate (2 equiv., 96.07 mg, 0.70 mmol) was added to an equimolar solution of 4-aminophenol (1 equiv., 37.93 mg, 0.35 mmol) and compound **17** (1 equiv., 100 mg, 0.35 mmol)

in DMF (1.5 mL). The resulting mixture was stirred at 100 ˚C for 2 h. After completion of the reaction, water and ethyl acetate were added to the crude reaction mixture. The aqueous layer was extracted with ethyl acetate. The combined organic phase was sequentially washed with deionized water and a saturated sodium chloride solution and then dried over anhydrous magnesium sulfate. After solvent removal under reduced pressure, the crude residue was subjected to purification by preparative HPLC (24% v/v H_2O+0.01%TFA/ACN+0.01%TFA). This process afforded the final product as a brown solid (53 mg, 48% yield). ^{1}H NMR (600 MHz, DMSO-d_6) δ = 10.36 (s, 1H), 7.23-7.20 (m, 4H), 6.51 (s, 1H), 5.78 (s, 1H), 2.12 (s, 3H). ^{13}C NMR (151 MHz, DMSO-d_6) δ = 164.40, 161.40, 149.29, 147.14, 138.39, 123.08, 121.40, 99.40, 95.94, 10.59. HRMS (ESI): m/z calculated for $C_{14}H_{14}ClN_6O$ $[M + H]^+$: 317.0913, Found: 317.0915.

4-((4-Chloro-6-((5-methyl-1*H*-pyrazol-3-yl)amino)pyrimidin-2-yl)oxy)benzaldehyde (IA174 or 4)

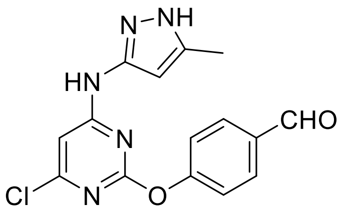

Synthesis was carried out by procedure adapted from literature.[1] The reaction afforded the pure product as a white powder (quantitative yield) after purification by preparative HPLC (34% v/v H_2O/ACN). ^{1}H NMR (700 MHz, DMSO-d_6) δ = 12.13-12.00 (m, 1H), 10.42-10.18 (m, 1H), 10.05 (s, 1H), 8.03 (m, 2H), 7.47 (m, 2H), 6.54 (s, 1H), 5.56 (s, 1H), 2.18-1.95 (m, 3H). ^{13}C NMR (176 MHz, DMSO-d_6) δ = 192.08, 176.46, 164.05, 161.22, 158.30, 157.45, 146.95, 138.15, 133.60, 131.35, 123.21, 99.80, 95.80, 10.47. HRMS (ESI): m/z calculated for $C_{15}H_{12}ClN_5O_2$ $[M + H]^+$: 330.0753, Found: 330.0749.

4-((4-Chloro-6-((5-methyl-1*H*-pyrazol-3-yl)amino)pyrimidin-2-yl)oxy)benzamide (IA167 or 5)

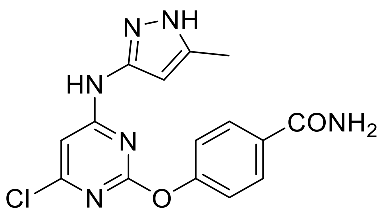

Synthesized by following the reported literature procedure and spectroscopic data were consistent with those reported in the literature.[1] 1:1 rotamers ^{1}H NMR (400 MHz, DMSO-d_6) δ = 11.97 (s, 1H), 10.36 (s, 1H), 8.02, 7.38 (two s, total 2H, two rotamers), 7.99, 7.30 (two m, total 4H, two rotamers), 7.73 (d, *J* = 8.6 Hz, 0.50 H, rotamer 1), 6.77 (d, *J* = 8.6 Hz, 0.50 H, rotamer 2), 6.52 (s, 0.50 H, rotamer 1), 5.52 (s, 0.50 H, rotamer 2), 2.22-1.97 (m, 3H).

6-Chloro-*N*-(5-methyl-1*H*-pyrazol-3-yl)-2-(4-(methylsulfonyl)phenoxy)pyrimidin-4-amine (IA168 or 6)

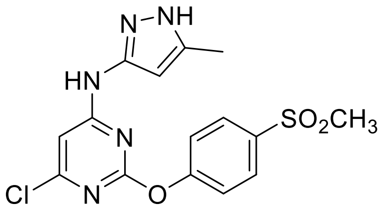

Synthesized by following the reported literature procedure and spectroscopic data were consistent with those reported in the literature.[1] ^{1}H NMR (500 MHz, DMSO-d_6) δ = 12.15-12.00 (m, 1H), 10.43 – 10.19 (m, 1H), 8.02 (m, 2H), 7.52 (d, *J* = 8.4 Hz, 2H), 6.56 (s, 1H), 5.62(s, 1H), 3.27 (s, 3H), 2.07 (s, 3H).

4-((4-Chloro-6-((5-methyl-1*H*-pyrazol-3-yl)amino)pyrimidin-2-yl)oxy)benzenesulfonamide (IA 169 or 7)

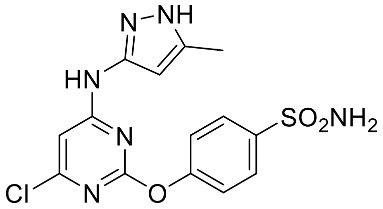

Synthesized by following the reported literature procedure and spectroscopic data were consistent with those reported in the literature.[1] ^{1}H NMR (500 MHz, DMSO-d_6) δ = 12.00 (s, 1H), 10.41 (s, 1H), 7.90 (d, *J* = 8.0, 2H), 7.44 – 7.42 (m, 4H), 6.55 (s, 1H), 5.69 (s,1H), 2.07 (s, 3H).

General procedure I for the synthesis of compounds (8-11)

Compound **3**, bearing a 4-aminophenol moiety, was reacted with the corresponding boronic acid derivative and glyoxylic acid monohydrate as the carbonyl source to facilitate the Petasis reaction.

The three components were dissolved in hexafluoroisopropanol (HFIP 0.1 M) and stirred at ambient temperature in the presence of 3Å molecular sieves. Progress of the reaction was monitored by TLC and LC-MS. Upon completion, the solvent was removed under reduced pressure, and the crude product was purified via preparative HPLC using a suitable acetonitrile-water gradient.

2-((4-((4-Chloro-6-((5-methyl-1*H*-pyrazol-3-yl)amino)pyrimidin-2-yl)oxy)phenyl)amino)-2-phenylacetic acid (IA181 or 8)

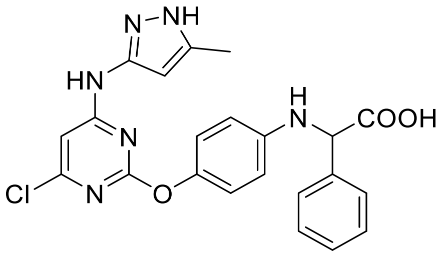

Synthesized according to General Procedure I, using compound **3** (1 equiv., 42 mg, 0.13 mmol), phenylboronic acid (1.2 equiv., 19.4 mg, 0.16 mmol), and glyoxylic acid monohydrate (1.2 equiv., 14.7 mg, 0.16 mmol). The reaction afforded the pure product as a brown powder (29.9 mg, 50% yield) after purification by preparative HPLC (42% v/v H_2O+0.01%TFA/ACN+0.01%TFA). ^{1}H NMR (600 MHz, DMSO-d_6) δ = 10.24 (s, 1H), 7.54 (d, *J* = 7.2 Hz, 2H), 7.37 (m, 2H), 7.31 (m, 1H), 6.89 (d, *J* = 9.0 Hz, 2H), 6.71 (d, *J* = 9.0 Hz, 2H), 5.76 (s, 1H), 5.08 (s, 1H), 3.10-3.05 (m, 1H), 2.09 (s, 3H). ^{13}C NMR (151 MHz, DMSO) δ = 172.95, 164.96, 161.38, 147.12, 144.57, 143.55, 138.56, 128.53, 127.87, 127.54, 124.23, 122.18, 113.42, 98.82, 95.84, 79.21, 60.18, 45.61, 10.50. HRMS (ESI): m/z calculated for $C_{22}H_{20}ClN_6O_3$ $[M + H]^+$: 451.1280, Found: 451.1276.

2-((4-((4-Chloro-6-((5-methyl-1*H*-pyrazol-3-yl)amino)pyrimidin-2-yl)oxy)phenyl)amino)-2-(2,4-dimethoxypyrimidin-5-yl)acetic acid (IA184 or 9)

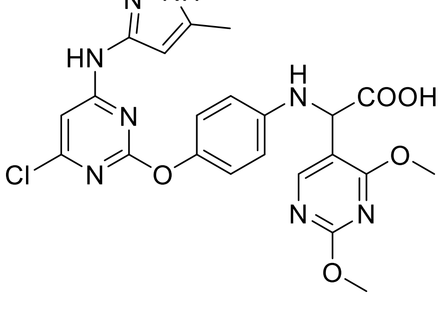

Synthesized according to General Procedure I, using compound **3** (1 equiv., 35 mg, 0.11 mmol), 2,4-dimethoxypyrimidin-5-boronic acid (1.2 equiv., 24.39 mg, 0.13 mmol), and glyoxylic acid monohydrate (1.2 equiv., 12.21 mg, 0.13 mmol). The reaction afforded the pure product as a white powder (35 mg, 35% yield) after purification by preparative HPLC (45% v/v H_2O/ACN). ^{1}H NMR (500 MHz, CD_3OD) δ = 8.37 (s, 1H), 6.99-6.97 (m, 2H), 6.78-6.77 (m, 2H), 6.65 (s, 1H), 5.81 (s, 1H), 5.30 (s, 1H), 4.13 (s, 3H), 4.04 (s, 3H), 2.16 (s, 3H). ^{13}C NMR (126 MHz, CD_3OD) δ 173.62, 170.98, 166.66, 165.83, 157.17, 146.36, 145.48, 125.26, 124.96, 123.72, 115.39, 114.08, 100.20, 97.48, 55.94, 55.35, 54.80,10.93. HRMS (ESI): m/z calculated for $C_{22}H_{22}ClN_8O_5$ [M + H]$^+$: 513.1397, Found: 513.1387.

2-((4-((4-Chloro-6-((5-methyl-1*H*-pyrazol-3-yl)amino)pyrimidin-2-yl)oxy)phenyl)amino)-2-(thiophen-2-yl)acetic acid (IA185 or 10)

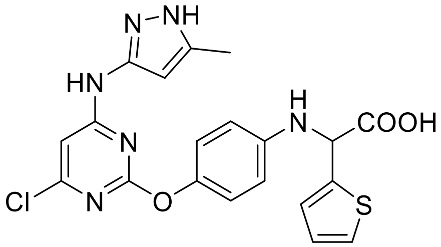

Synthesized according to General Procedure I, using compound **3** (1 equiv., 27 mg, 0.09 mmol), 2-thienylboronic acid (1.2 equiv., 9.41 mg, 0.10 mmol), and glyoxylic acid monohydrate (1.2 equiv., 13.09 mg, 0.10 mmol). The reaction afforded the pure product as a yellow powder (19 mg, 49 % yield) after purification by preparative HPLC (48% v/v H_2O/ACN). ^{1}H NMR (500 MHz, DMSO-d_6) δ = 12.03 (s, 1H), 10.27 (s, 1H), 7.47 (d, 1H, J = 5.0 Hz), 7.21 (m, 1H), 7.03-7.01 (m, 1H), 6.92-6.91 (m, 2H), 6.76 (d, J = 7.5 Hz, 2H), 6.46 (s, 1H), 5.76 (s, 1H), 5.35 (s, 1H), 2.08 (s, 3H). HRMS (ESI): m/z calculated for $C_{20}H_{18}ClN_6O_3S$ [M + H]$^+$: 457.0845, Found: 457.0845.

2-((4-((4-Chloro-6-((5-methyl-1*H*-pyrazol-3-yl)amino)pyrimidin-2-yl)oxy)phenyl)amino)-2-(3,5-dimethylisoxazol-4-yl)acetic acid (IA186 or 11)

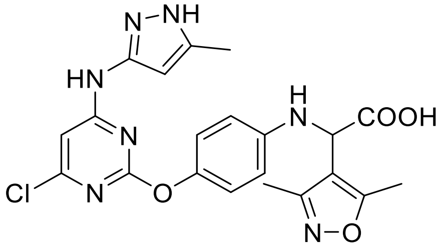

Synthesized according to General Procedure I, using compound **3** (1 equiv., 30 mg, 0.09 mmol), 3,5-dimethylisoxazol-4-yl-4-boronic acid (1.2 equiv., 16.02 mg, 0.11 mmol), and glyoxylic acid monohydrate (1.2 equiv., 10.46 mg, 0.11 mmol). The reaction afforded the pure product as a brown powder (15 mg, 33 % yield) after purification by preparative HPLC (44% v/v H_2O/ACN). ^{1}H NMR (600 MHz, CD_3OD) δ = 7.46-7.45 (m, 1H), 7.39-7.38 (m, 1H), 6.98-6.97 (m, 2H), 6.72-6.70 (m, 2H), 6.66 (s, 1H), 5.37 (s, 1H), 5.03 (s, 1H), 2.47 (s, 3H), 2.31 (s, 3H), 2.16 (s, 3H). ^{13}C NMR (151 MHz, CD_3OD) δ = 173.70, 168.86, 166.82, 160.79, 146.26, 145.91, 142.55, 125.19, 125.07, 123.81, 115.05, 112.89, 100.21, 99.67, 52.83, 11.46, 11.12, 10.62. HRMS (ESI): m/z calculated for $C_{21}H_{21}ClN_7O_4$ $[M + H]^+$: 470.1339, Found: 470.1348.

***N*-(5-Methyl-1*H*-pyrazol-3-yl)-6-(4-methylpiperazin-1-yl)-2-(4-nitrophenoxy)pyrimidin-4-amine (SH4 or 1)**

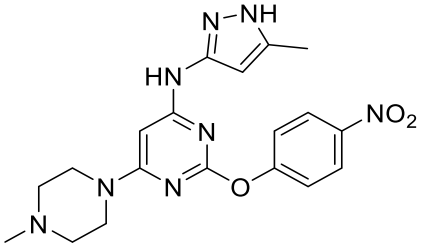

Synthesized by following the reported literature procedure and spectroscopic data were consistent with those reported in the literature.[2] ^{1}H NMR (500 MHz, DMSO-d_6) δ = 10.30 (s, 1H), 9.60 (s, 1H), 8.32-8.30 (m, 2H), 7.46-7.43 (m, 2H), 6.33 (s, 1H), 5.66 (s, 1H), 4.19 (m, 2H), 3.48 (m, 2H), 3.22-3.05 (m, 4H), 2.82 (s, 3H), 2.07 (s, 3H). ^{13}C NMR (126 MHz, DMSO-d_6) δ 163.75, 163.41, 161.75, 158.62, 148.06, 143.93, 138.65, 125.30, 122.68, 95.22, 80.33, 51.75, 42.18, 41.10, 10.50. HRMS (ESI): m/z calculated for $C_{19}H_{23}N_8O_3$ $[M + H]^+$: 411.1815 , Found: 411.1888.

2-(4-(6-((5-Methyl-1*H*-pyrazol-3-yl)amino)-2-(4-sulfamoylphenoxy)pyrimidin-4-yl)piperazin-1-yl)-2-phenylacetic acid (AK177 or 2)

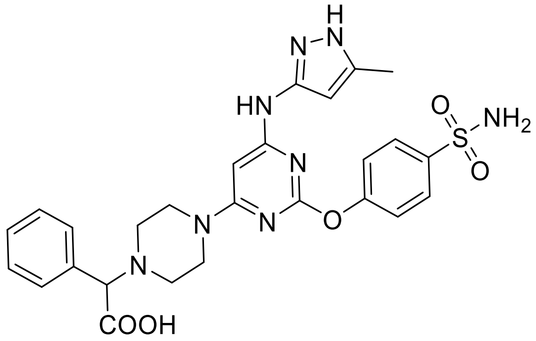

Compound **7** (1 equiv., 35 mg, 0.09 mmol**)** was dissolved in 1.5 mL of 1,4-dioxane, and to it piperazine (6.5 equiv., 51.46 mg, 0.60 mmol) was added and stirred under refluxing condition until complete conversion monitored by LC-MS. The crude reaction mixture was allowed to come to room temperature, to which was added saturated aq. Sodium bicarbonate solution. The aqueous layer was extracted with ethyl acetate. The resultant organic phase was evaporated to yield the crude product (**21**), which was directly used in the next step.

Intermediate (**21**) (1.0 equiv., 33.00 mg, 0.08 mmol) was reacted with glyoxylic acid monohydrate (1.2 equiv., 8.47 mg, 0.09 mmol) and phenylboronic acid (1.2 equiv., 11.22 mg, 0.09 mmol) in HFIP (0.1 M), with 3Å molecular sieves. The mixture was stirred at room temperature, and after completion, the solvent was removed under vacuum. The crude residue was purified by preparative HPLC using a gradient of water and acetonitrile (23% v/v H_2O/ACN), yielding compound **2** as a white solid (32 mg, 75% yield). ^{1}H NMR (500 MHz, CD_3OD) δ = 7.96-7.94 (m, 2H), 7.74-7.72 (m, 1H), 7.57-7.55 (m, 2H), 7.47-7.46 (m, 3H), 7.31 (d, *J* = 8.5 Hz, 2H), 6.88-6.86 (m, 1H), 5.75 (s, 1H), 4.52 (s, 1H), 3.75 (s, 4H), 3.29 (s, 4H), 2.97-2.95 (m, 2H), 2.19 (s, 3H) ^{13}C NMR (126 MHz, CD_3OD) δ ^{13}C NMR (126 MHz, CD_3OD) δ = 165.50, 163.66, 162.44, 157.59, 141.49, 134.86, 133.28, 131.03, 130.64, 130.38, 129.35, 128.81, 123.53, 116.31, 96.47, 80.88, 75.64, 51.82, 49.51, 49.34, 49.17, 49.00, 48.83, 48.66, 48.49, 42.78, 11.10. HRMS (APCI): m/z calculated for $C_{26}H_{29}N_8O_5S$ $[M + H]^+$: 565.1977, Found: 565.1986.

LC-MS Spectra

IA164 (16) (C-18 column_10-100% (H_2O/ACN) _6 min)

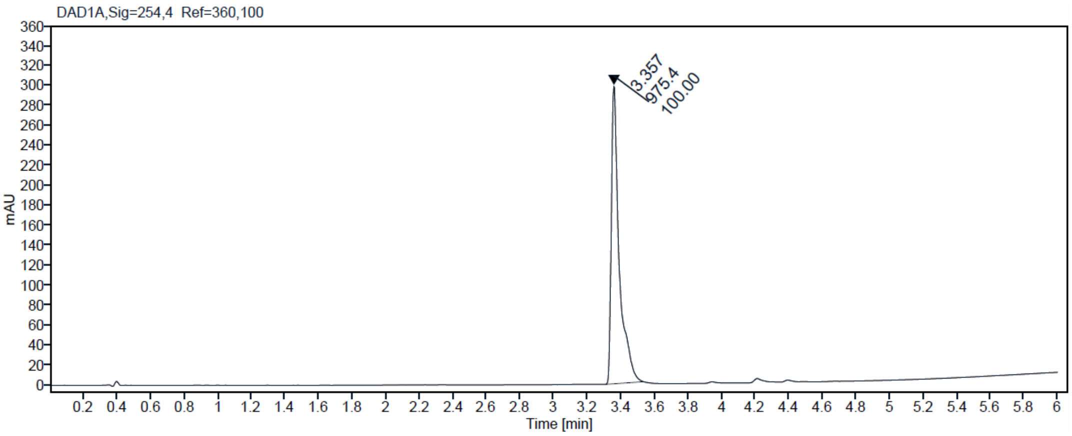

Signal positive-mode ESI-MS1 scan

Peak RT 3.413

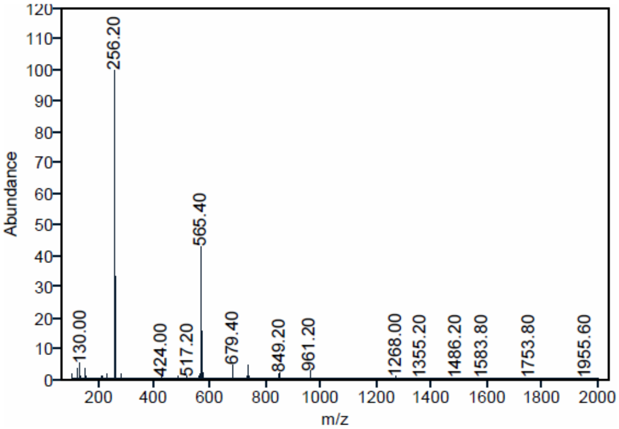

IA165 (17) (C-18 column_10-100% (H_2O/ACN) _6 min)

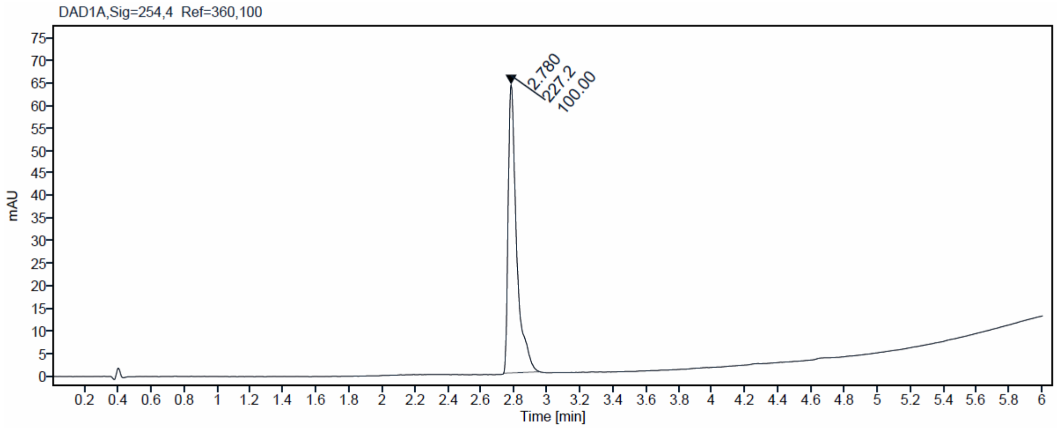

Signal positive-mode ESI-MS1 scan

Peak RT 2.830

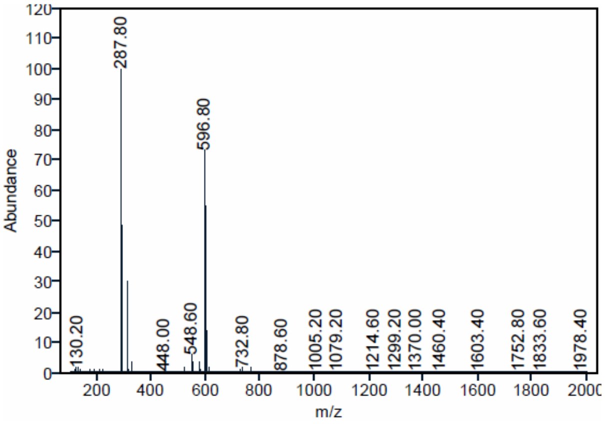

IA180 (3) (C-18 column_10-100% (H_2O/ACN) _6 min)

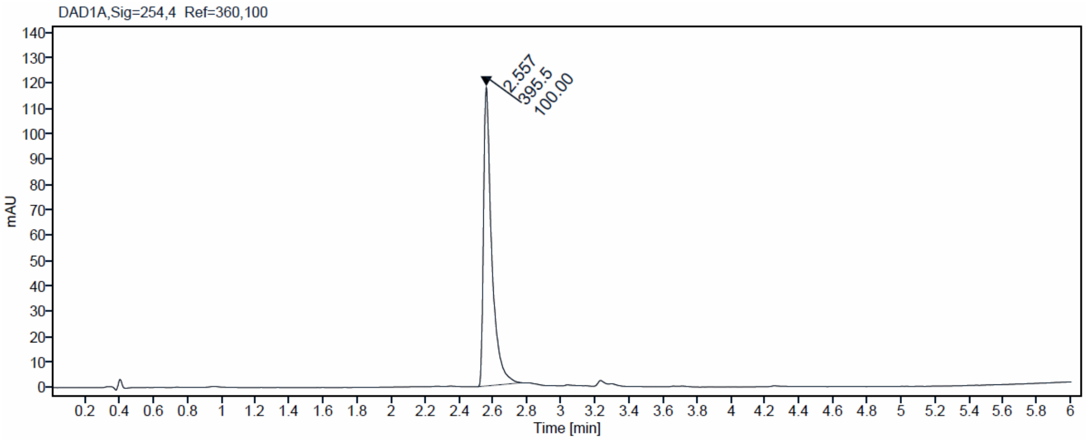

Signal positive-mode ESI-MS1 scan

Peak RT 2.623

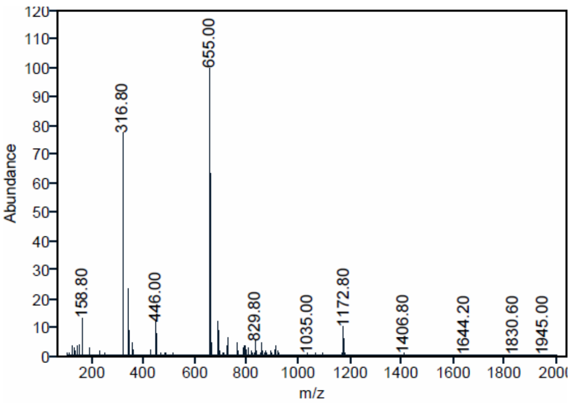

IA174 (4) (C-18 column_10-100% (H_2O/ACN) _6 min)

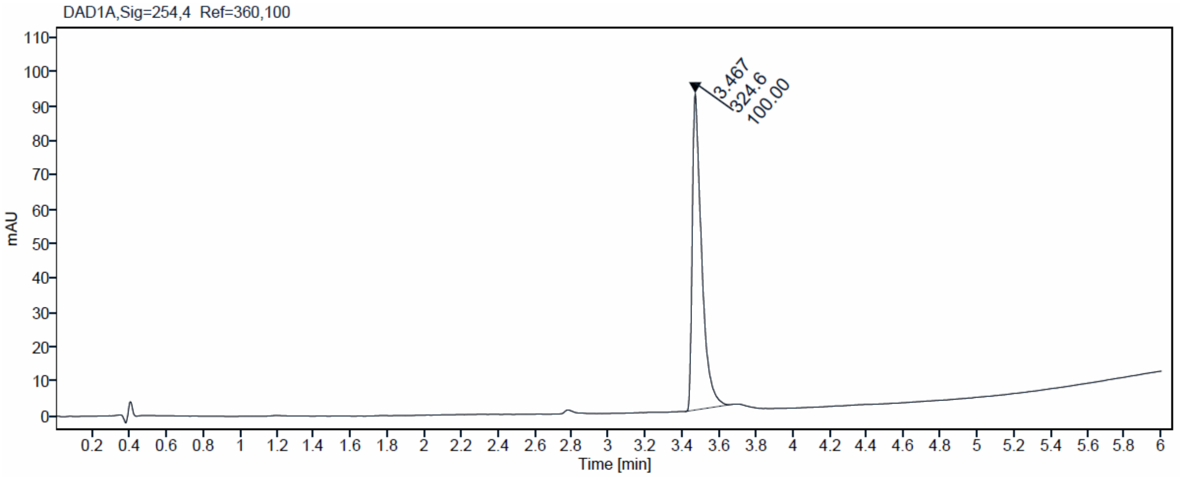

Signal positive-mode ESI-MS1 scan

Peak RT 3.525

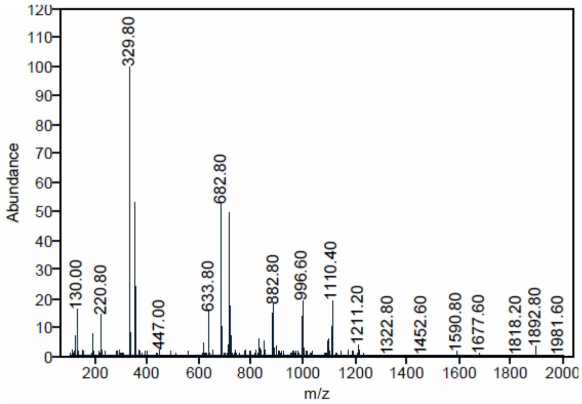

IA167 (5) (C-18 column_10-100% (H_2O/ACN) _6 min)

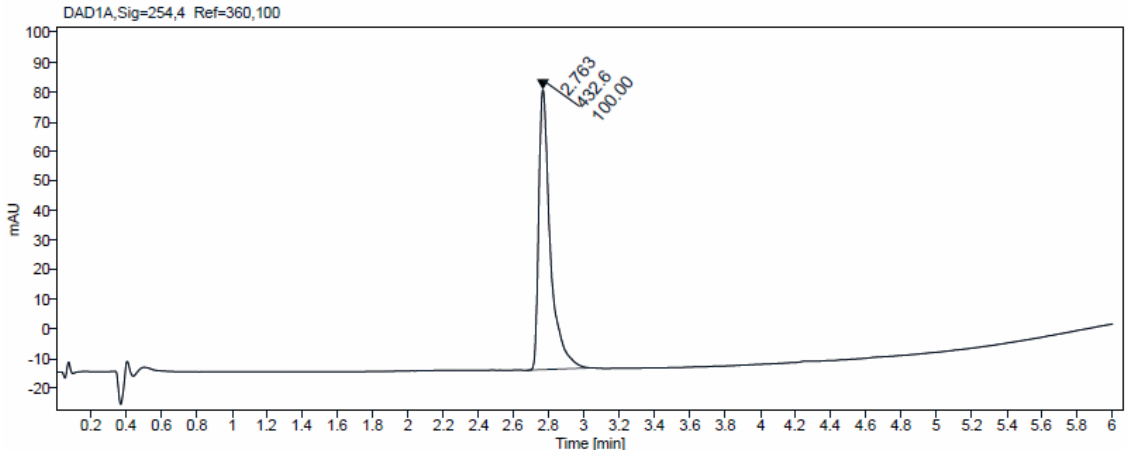

Signal positive-mode ESI-MS1 scan

Peak RT 2.851

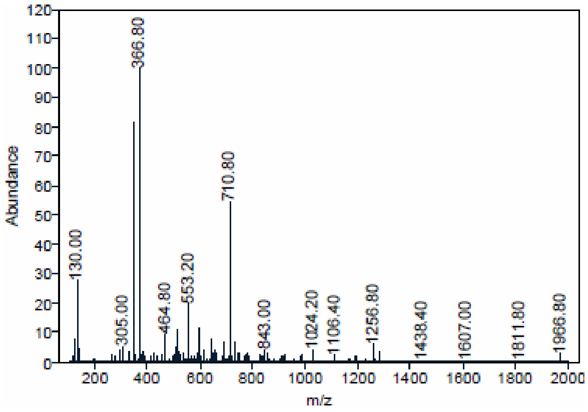

IA168 (6) (C-18 column_10-100% (H_2O/ACN) _6 min)

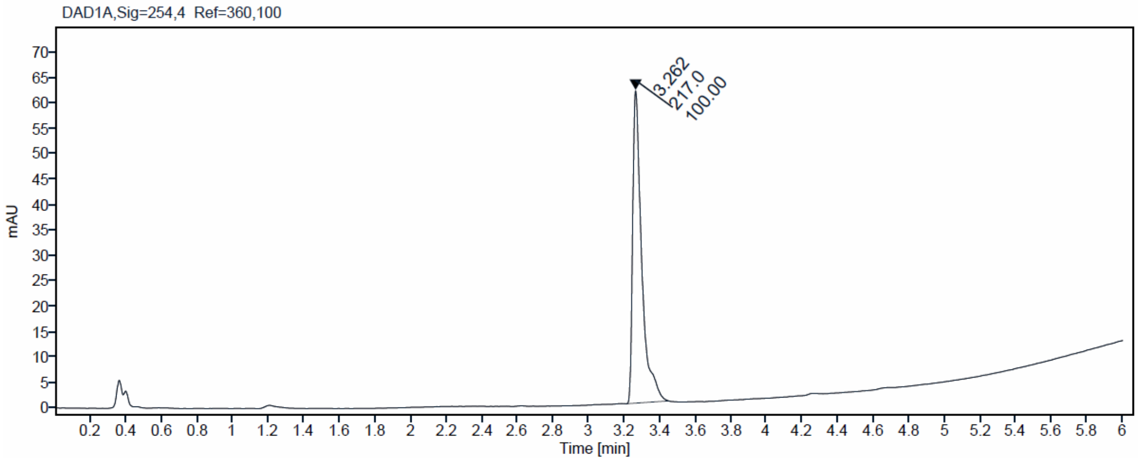

Signal positive-mode ESI-MS1 scan

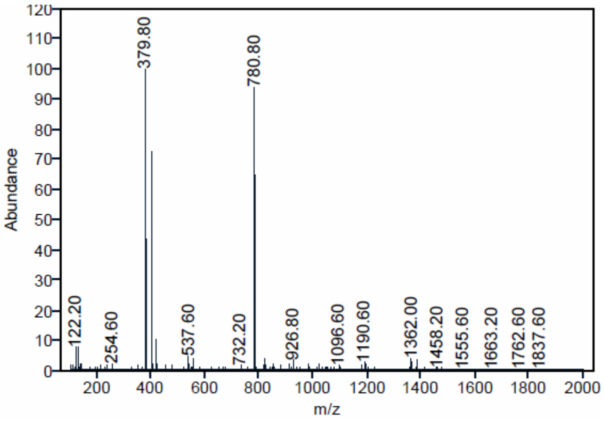

IA169 (7) (C-18 column_10-100% (H_2O/ACN) _6 min)

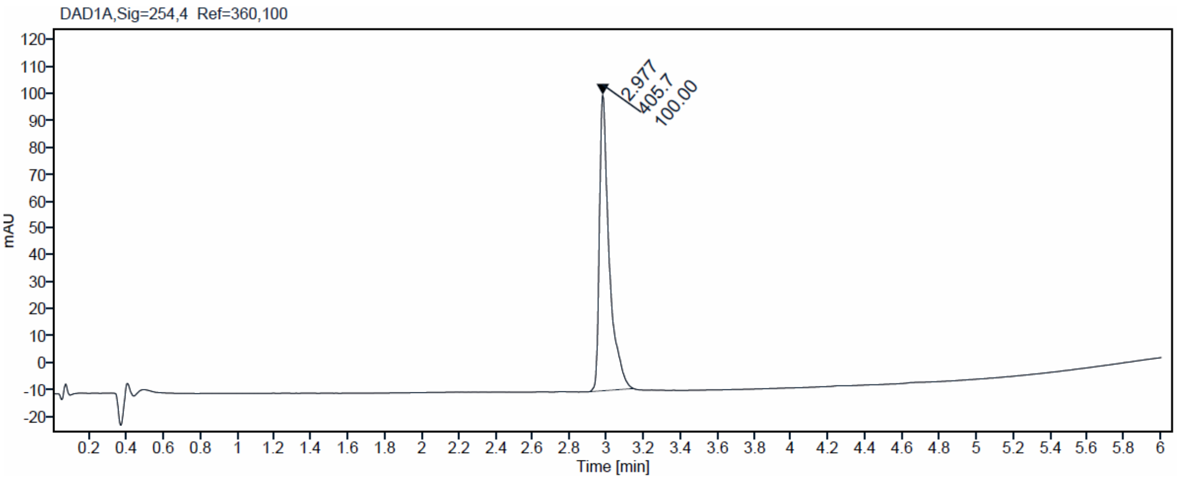

Signal positive-mode ESI-MS1 scan

Peak RT 3.042

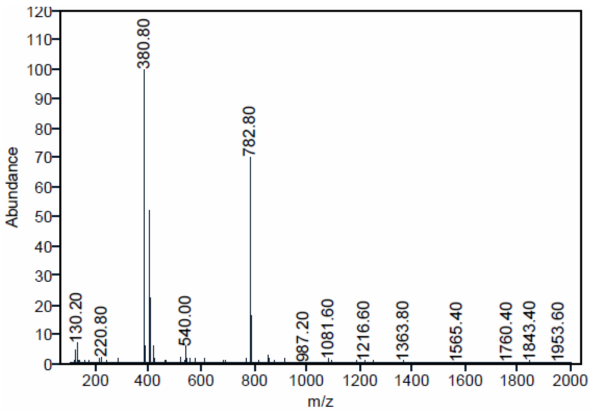

IA181(8) (C-18 column_10-100% (H_2O/ACN) _6 min)

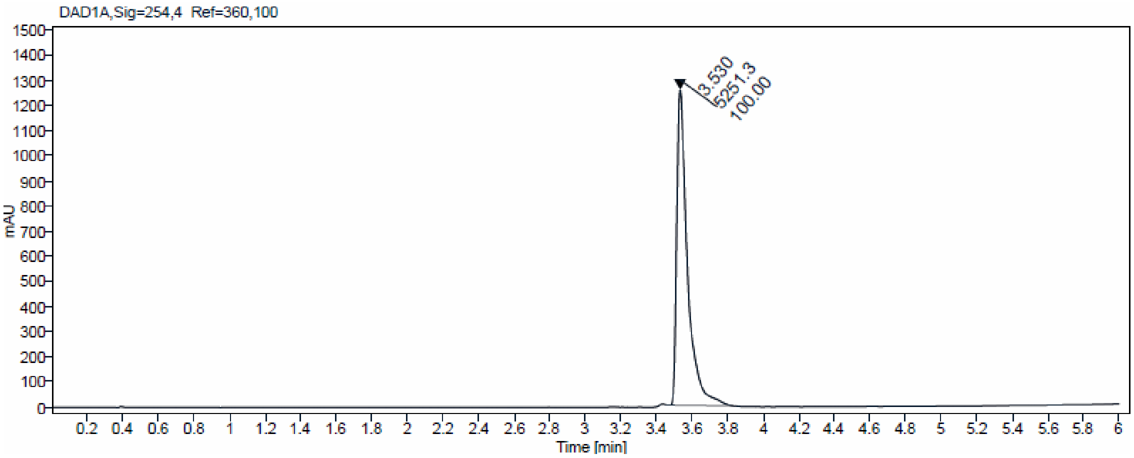

Signal positive-mode ESI-MS1 scan

Peak RT 3.605

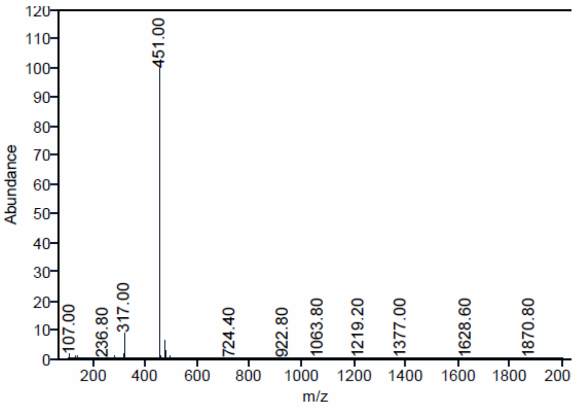

IA184 (9) (C-18 column_10-100% (H_2O/ACN) _6 min)

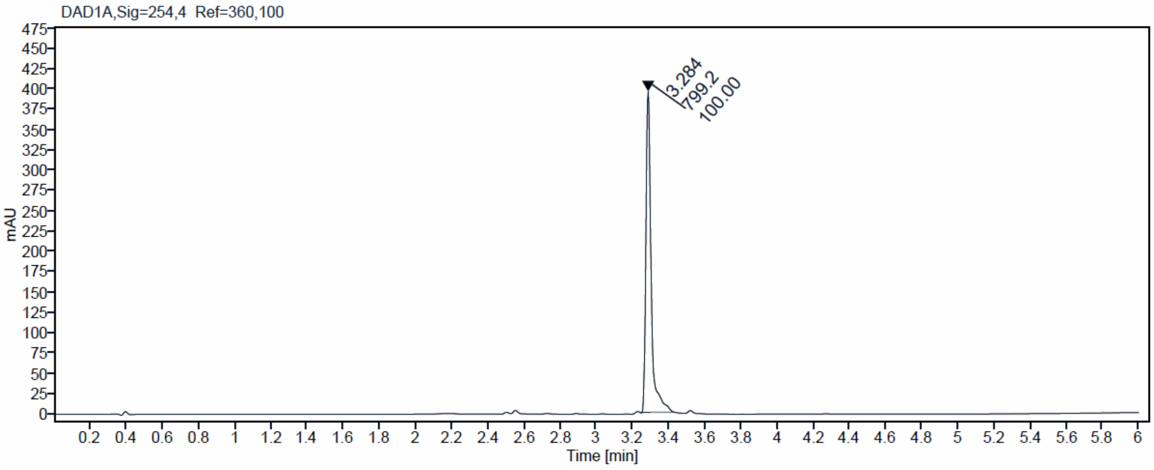

Signal positive-mode ESI-MS1 scan

Peak RT 3.348

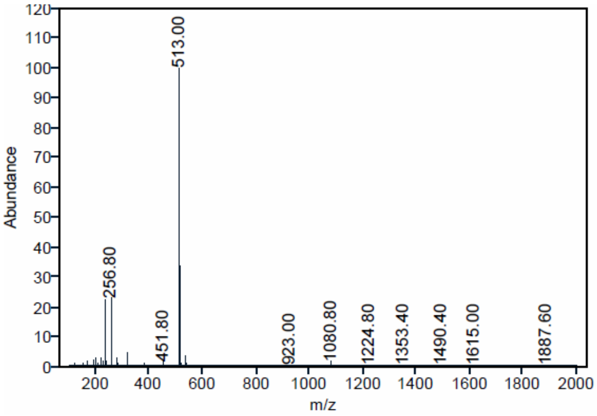

IA185 (10) (C-18 column_10-100% (H_2O/ACN) _6 min)

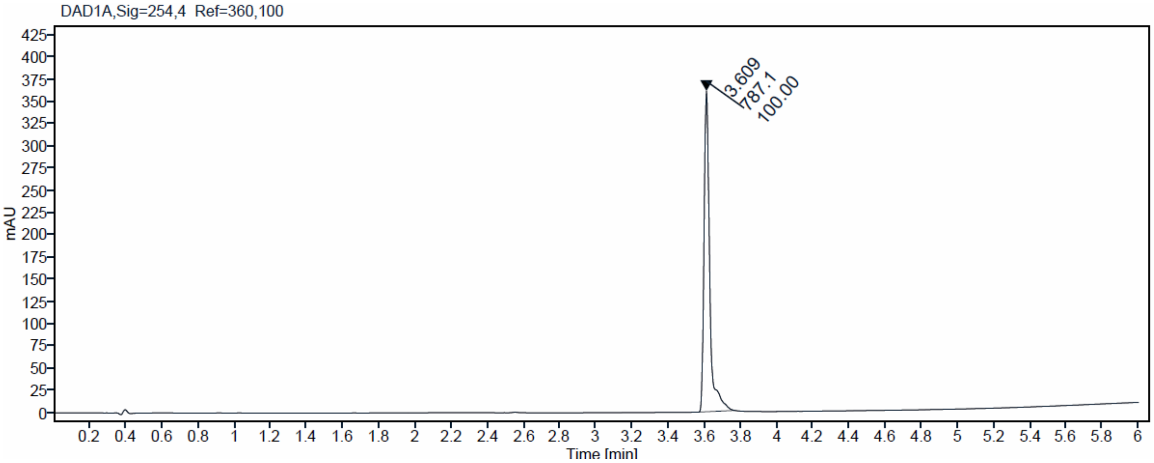

Signal positive-mode ESI-MS1 scan

Peak RT 3.660

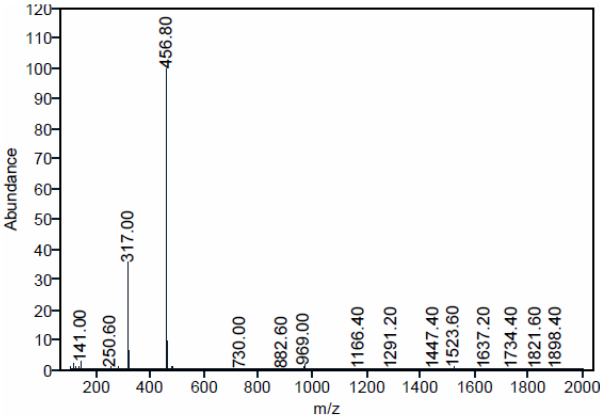

IA186 (11) (C-18 column_10-100% (H_2O/ACN) _6 min)

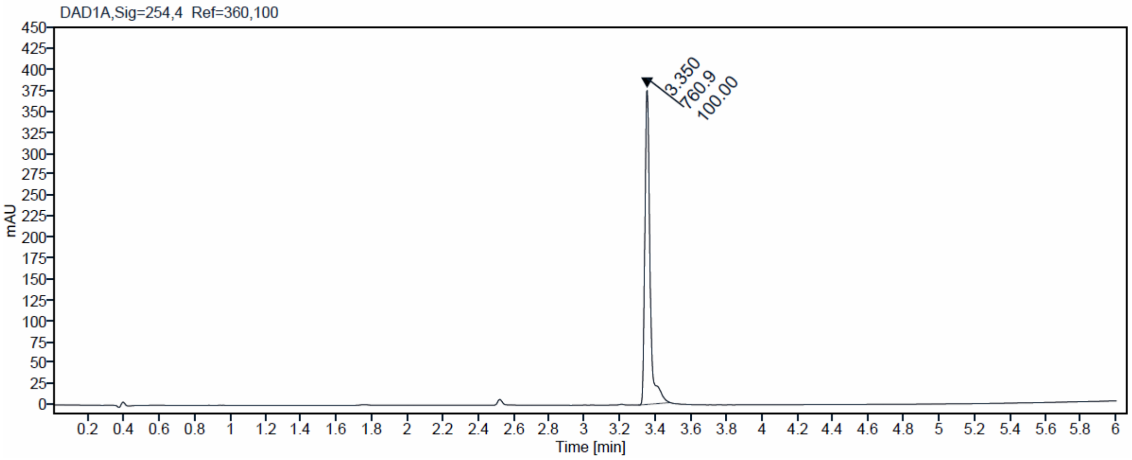

Signal positive-mode ESI-MS1 scan

Peak RT 3.414

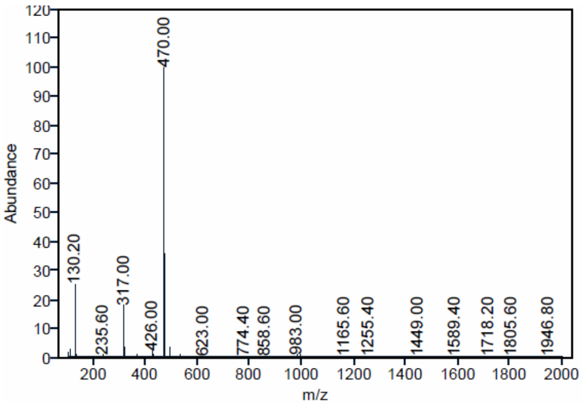

SH4 (1) (C-18 column_10-100% (H_2O/ACN) _6 min)

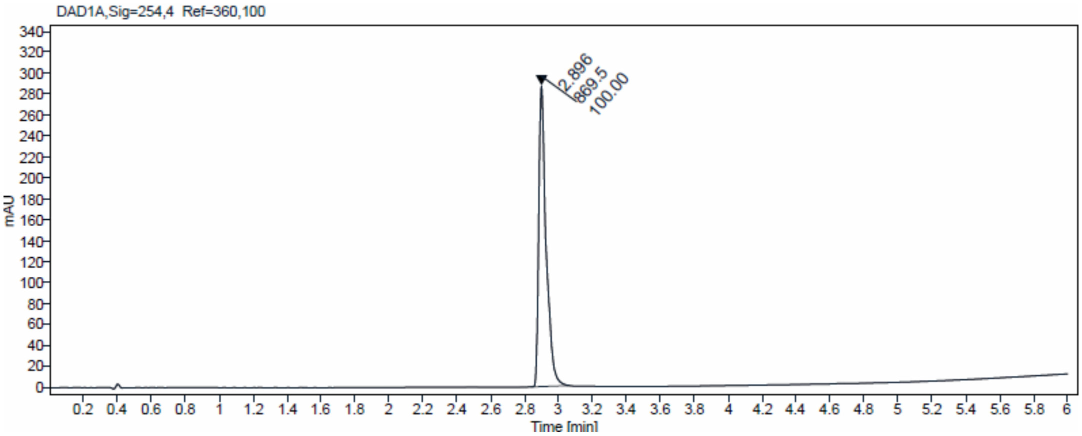

Signal positive-mode ESI-MS1 scan

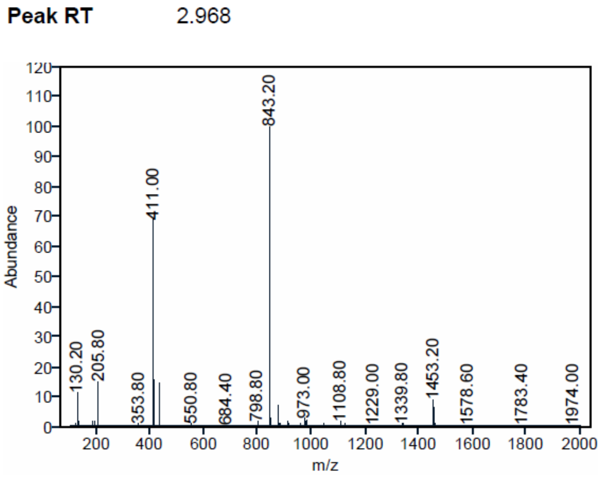

AK177 (2) (C-18 column_10-100% (H_2O/ACN) _6 min)

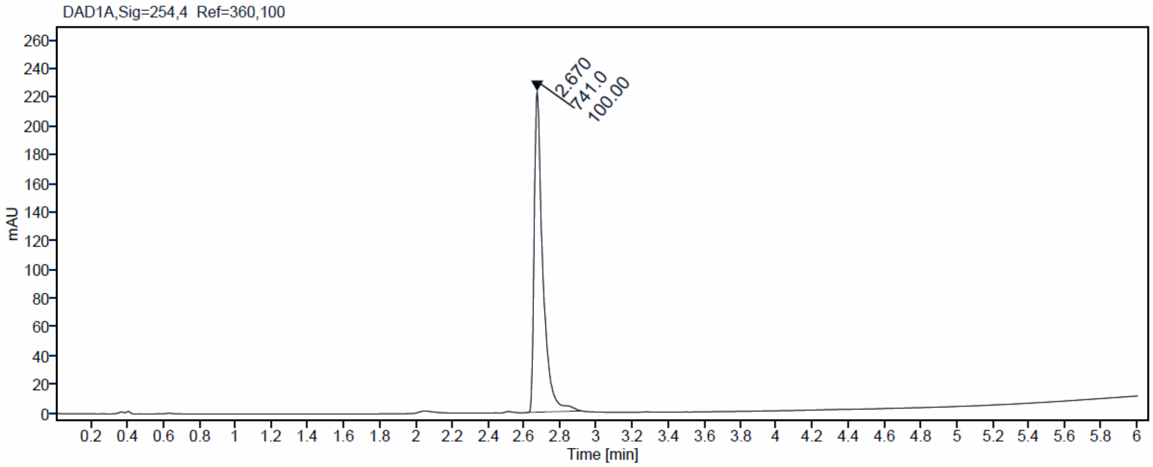

Signal positive-mode ESI-MS1 scan

Peak RT 2.740

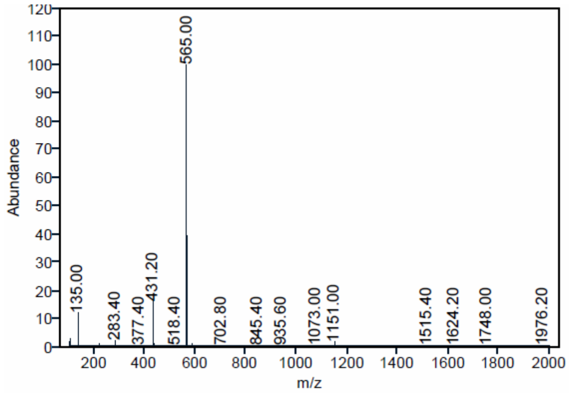

NMR Spectra

^{1}H NMR IA164 (16) (DMSO-d_6, 500 MHz)

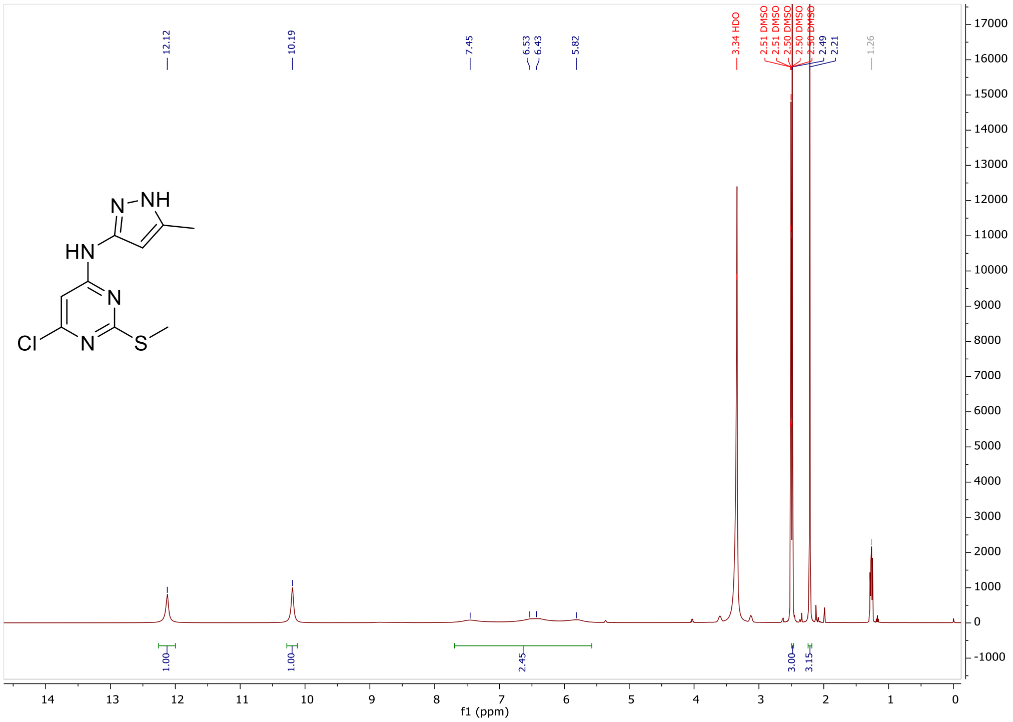

^{1}H NMR IA165 (17) (DMSO-d_6, 500 MHz)

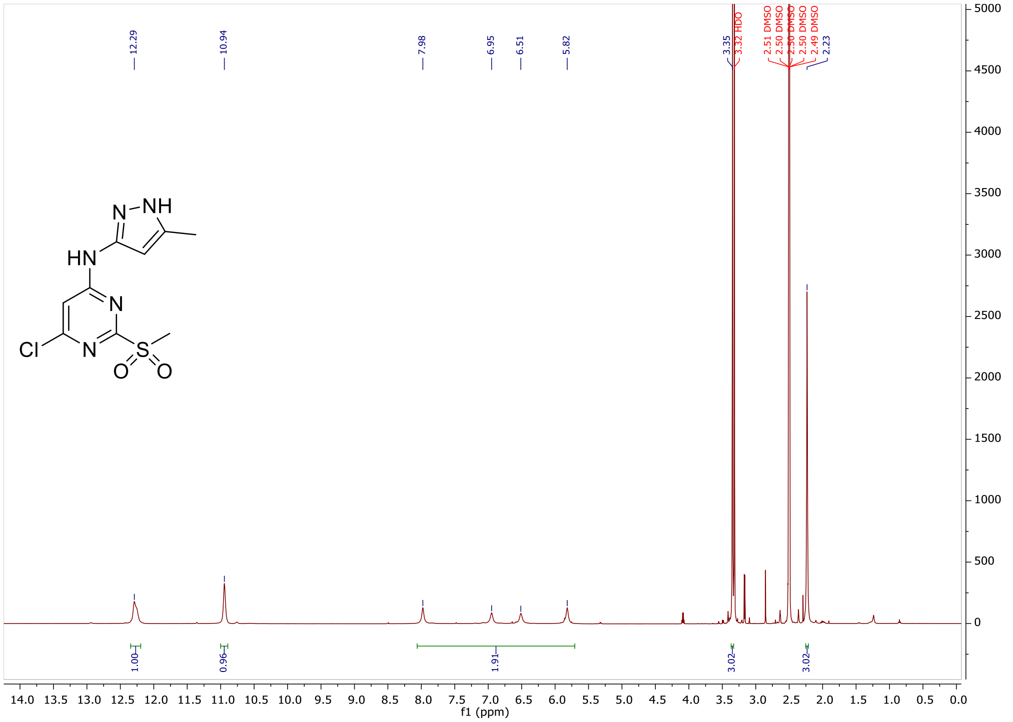

^{1}H NMR IA180 (3) (DMSO-d_6, 600 MHz)

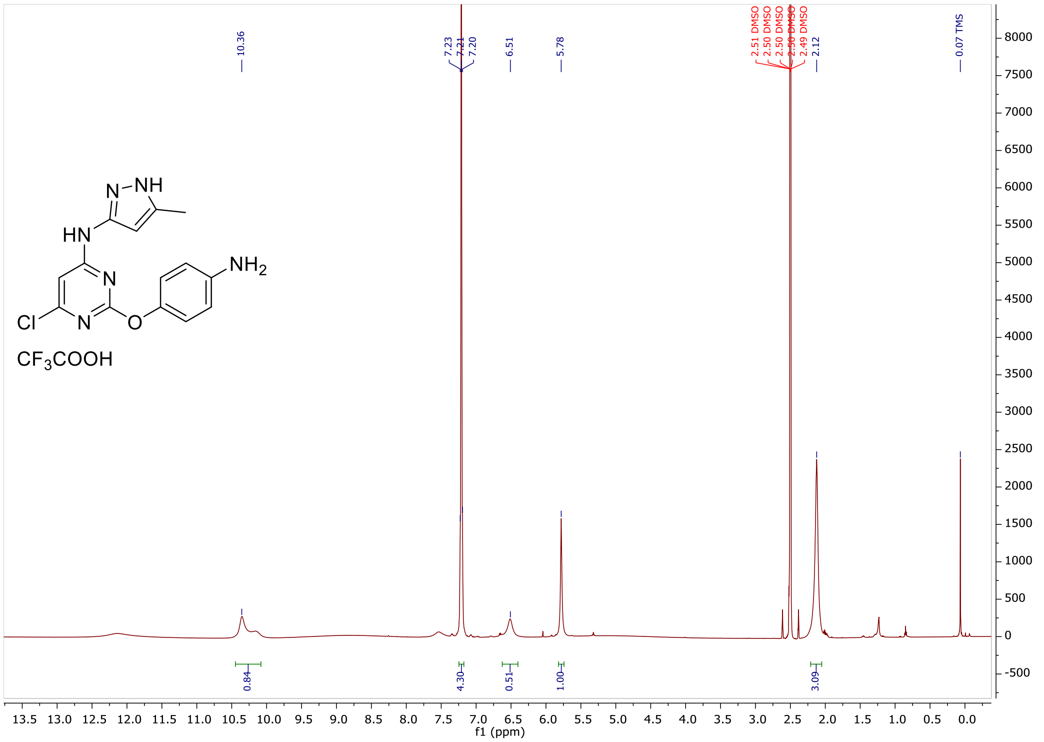

^{13}C NMR IA180 (3) (DMSO-d_6, 151 MHz)

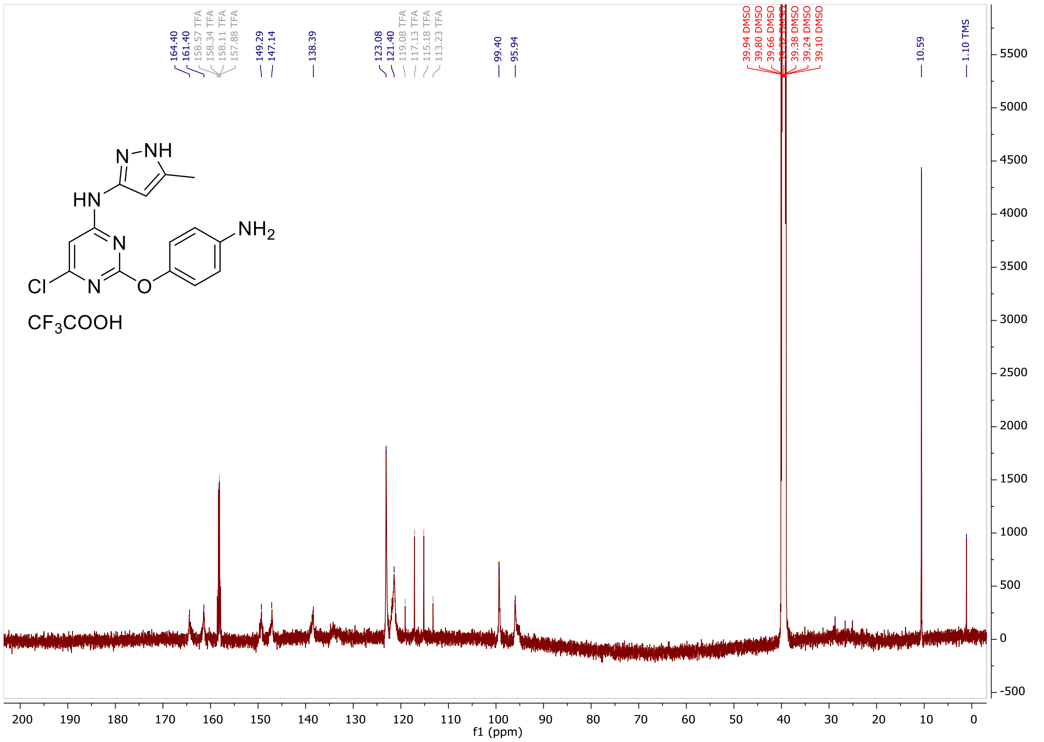

^{1}H NMR IA174 (4) (DMSO-d_6, 700 MHz)

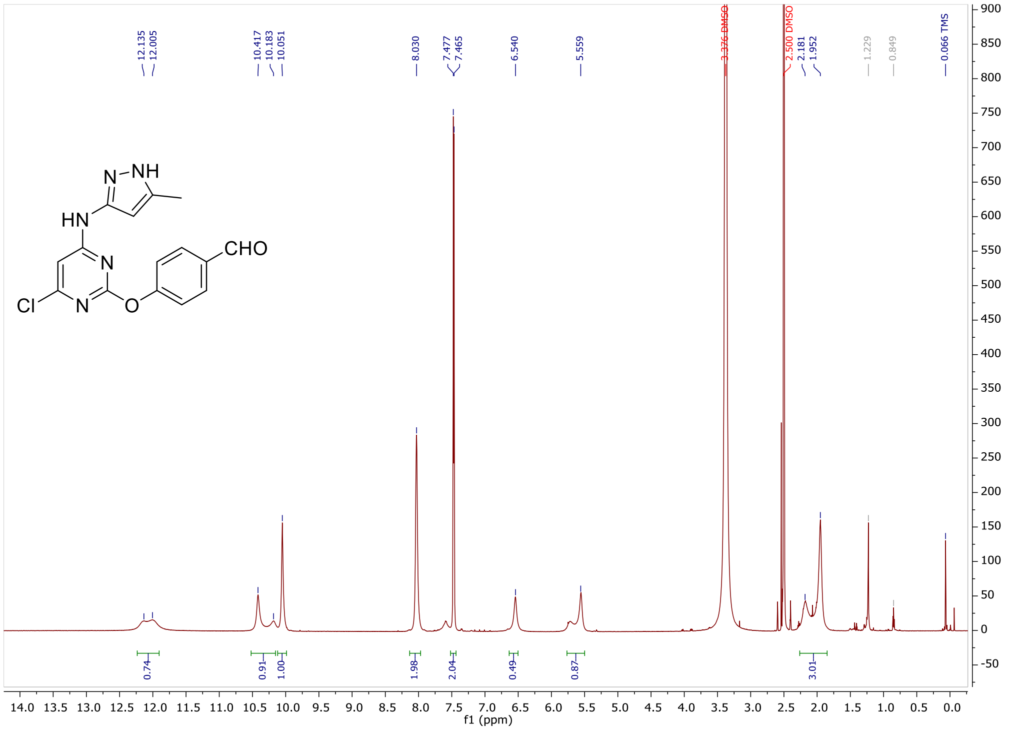

^{13}C NMR IA174 (4) (DMSO-d_6, 176 MHz)

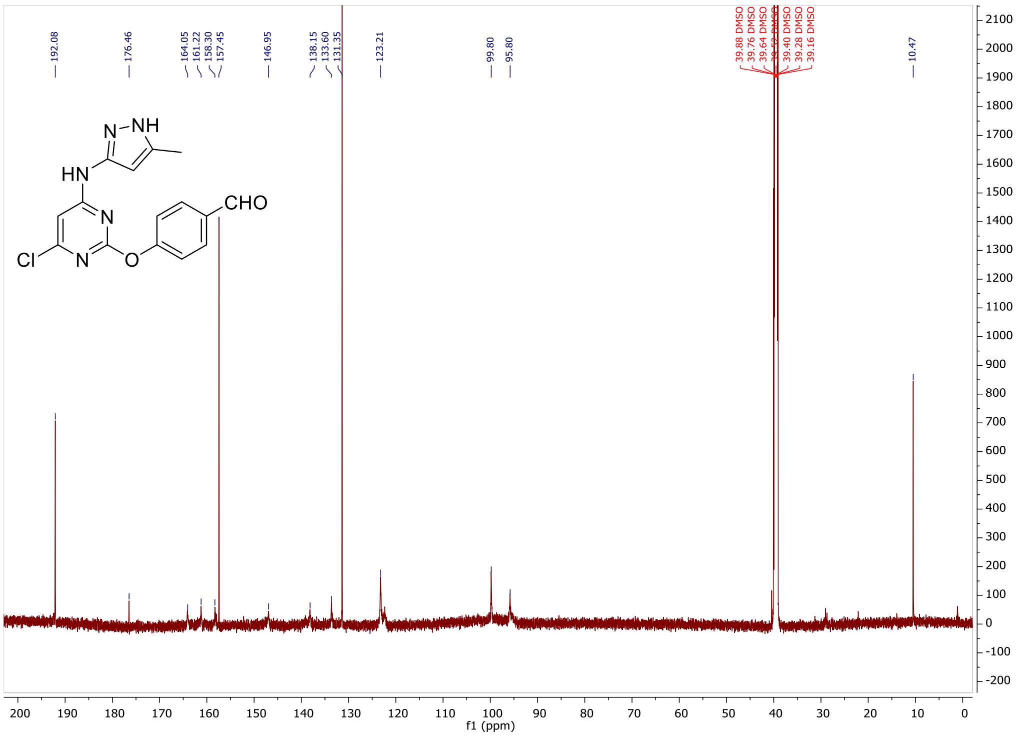

^{1}H NMR IA167 (5) (DMSO-d_6, 400 MHz)

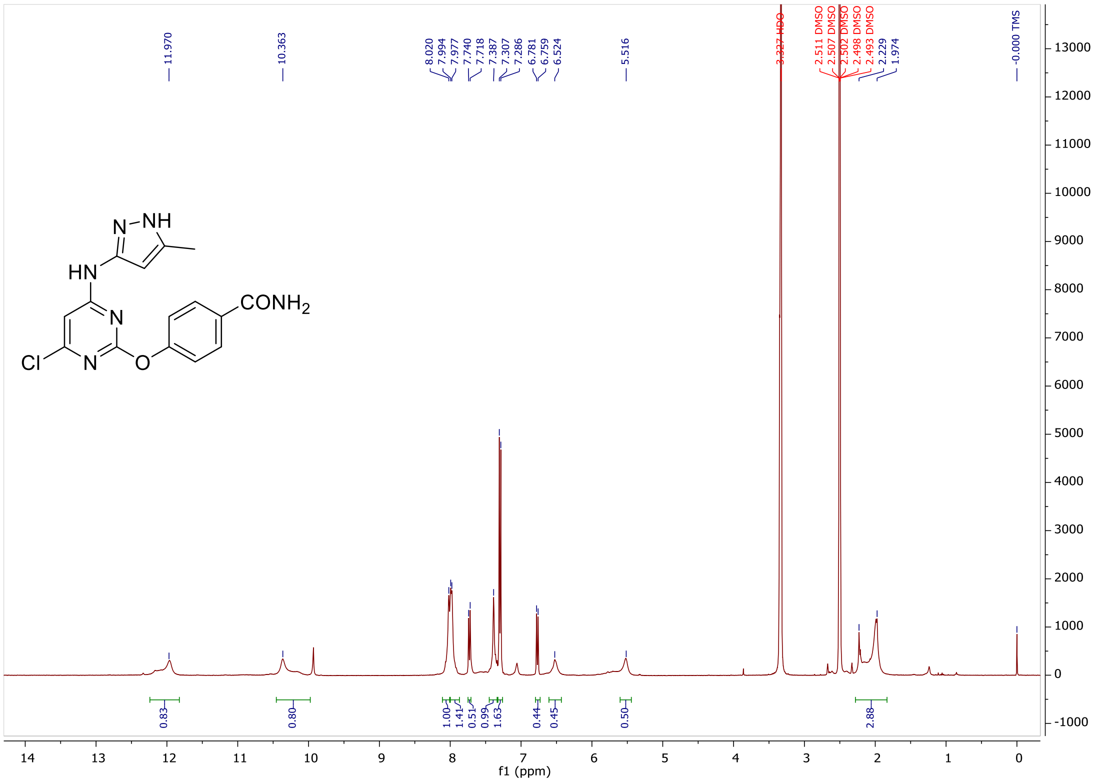

^{1}H NMR IA168 (6) (DMSO-d_6, 700 MHz)

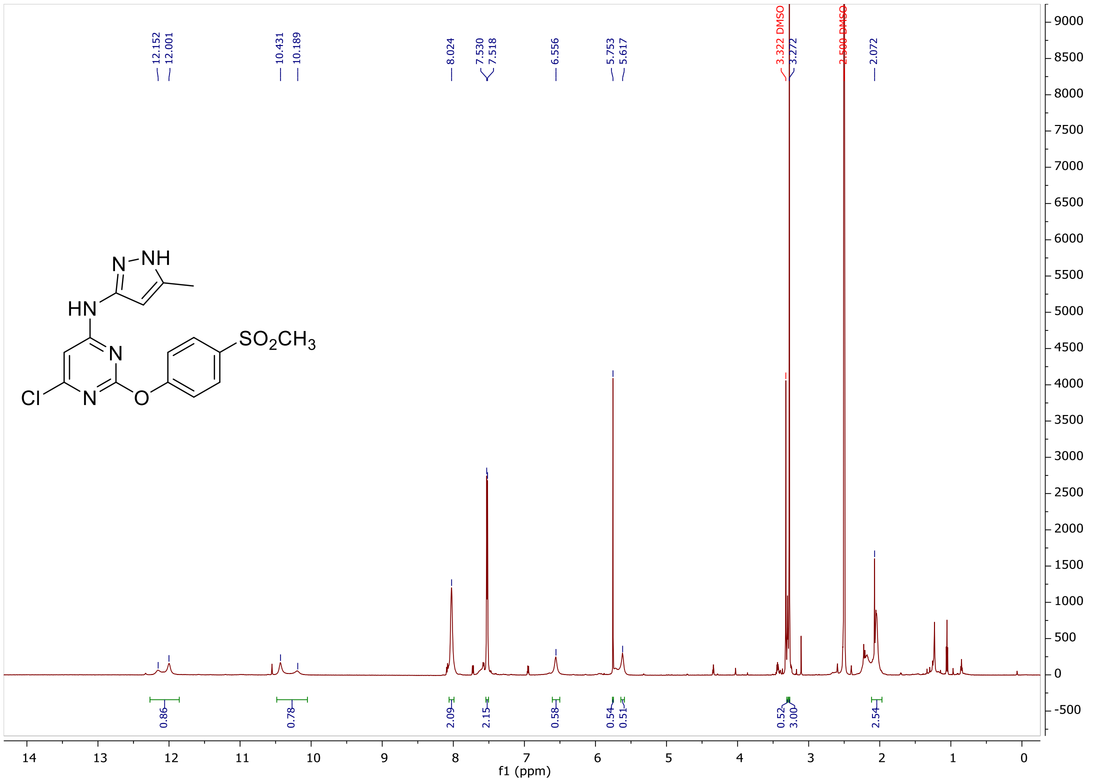

^{1}H NMR IA169 (7) (DMSO-d_6, 700 MHz)

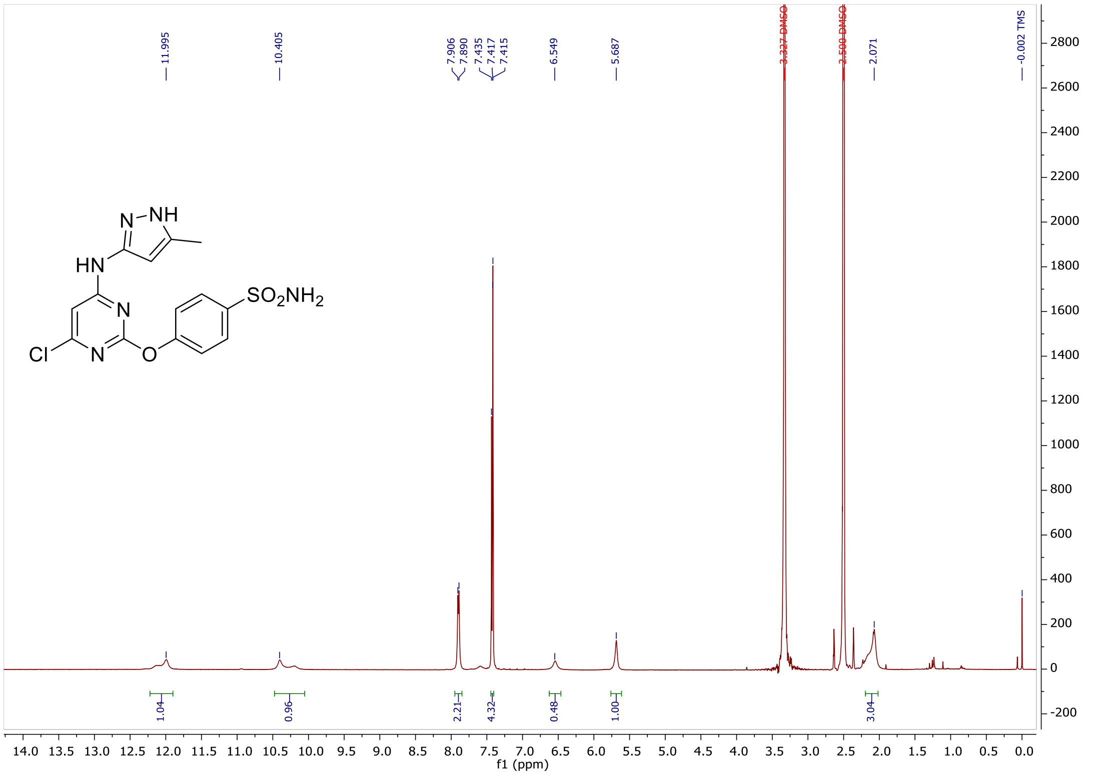

^{1}H NMR IA181 (8) (DMSO-d_6, 600 MHz)

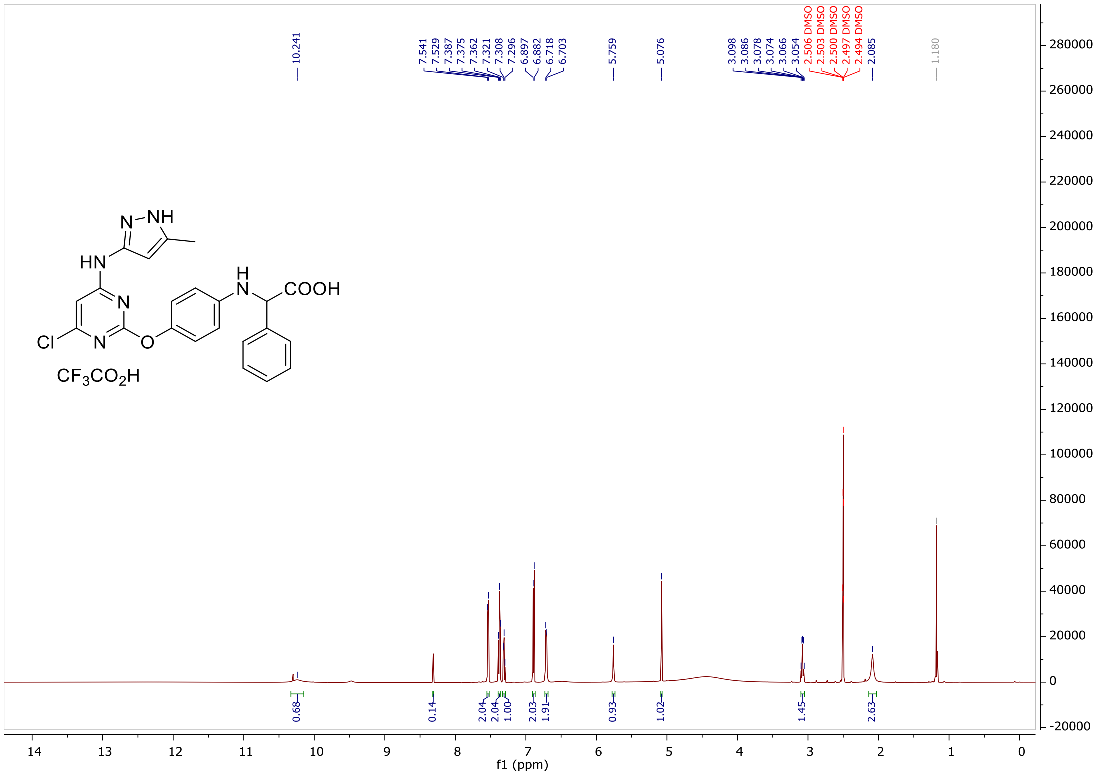

^{13}C NMR IA181 (8) (DMSO-d_6, 151 MHz)

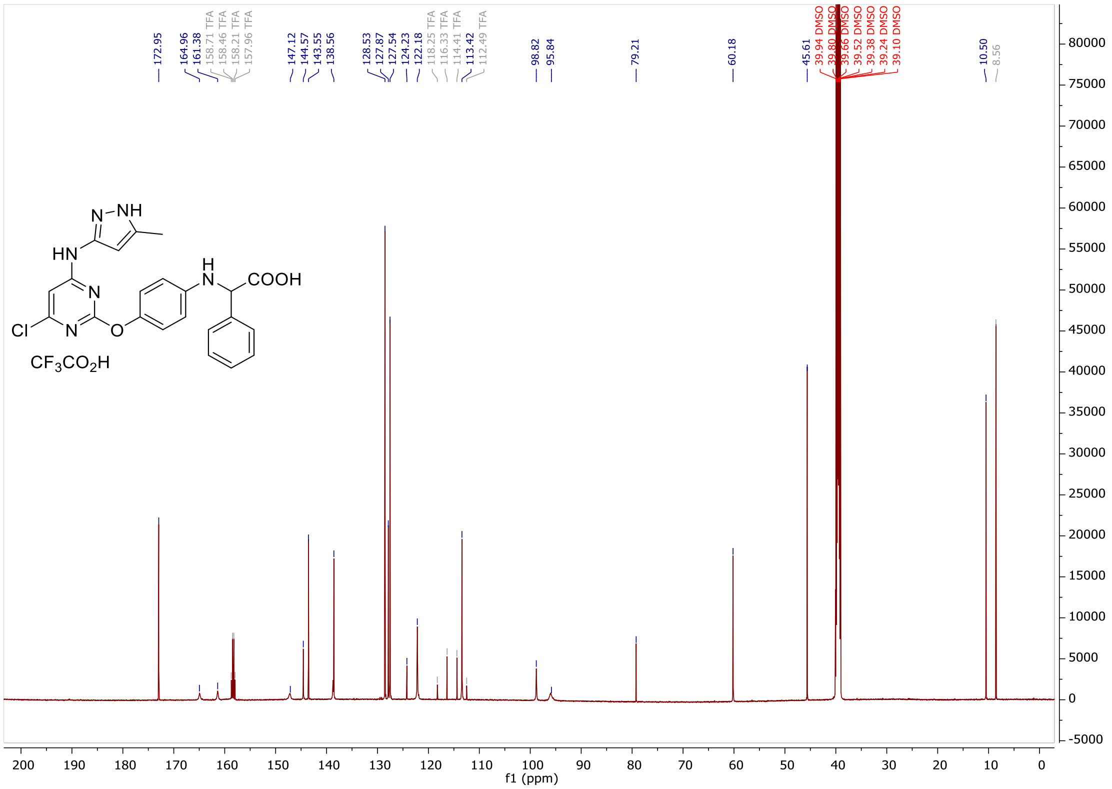

^{1}H NMR 184 (9) (CD_3OD, 500 MHz)

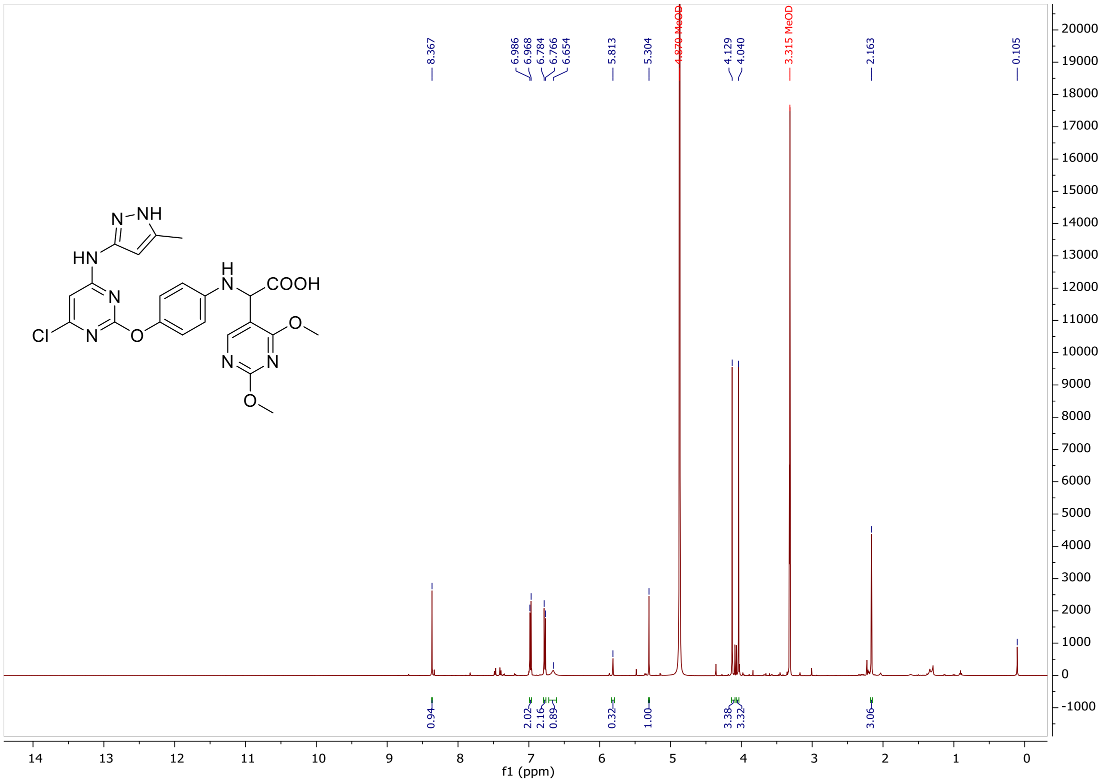

^{13}C NMR 184 (9) (CD_3OD, 126 MHz)

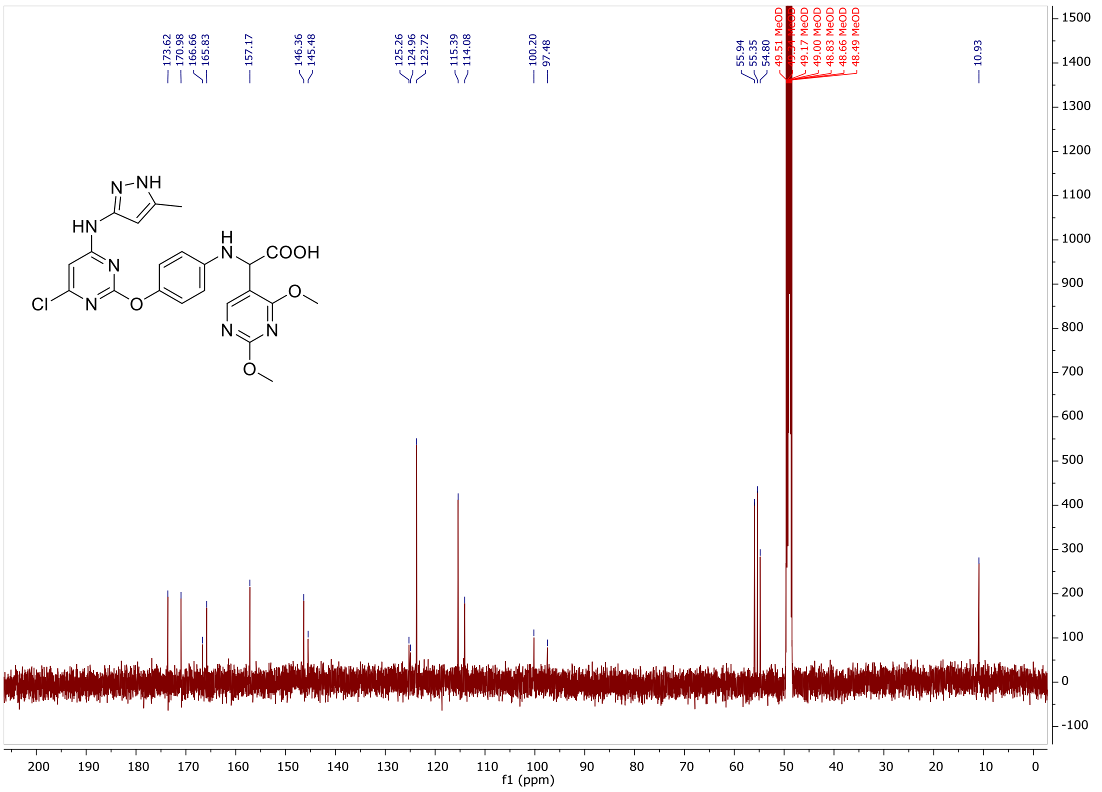

^{1}H NMR IA185 (10) (DMSO-d$_6$, 500 MHz)

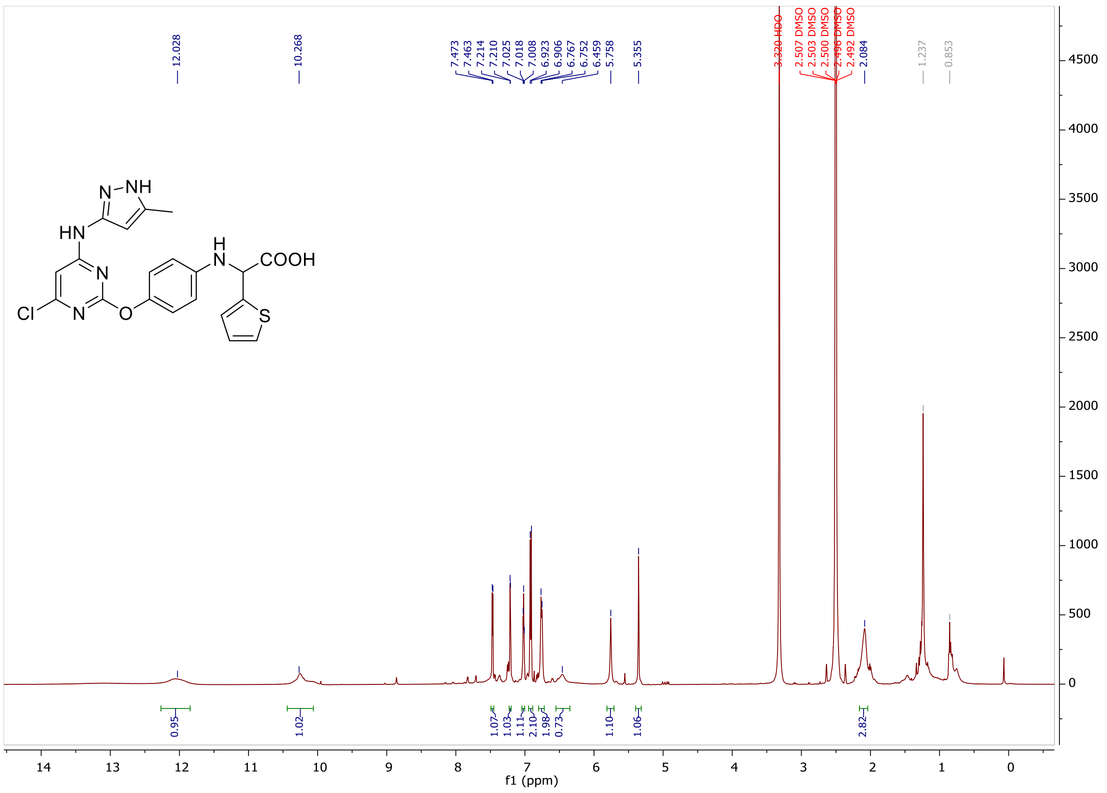

^{1}H NMR IA186 (11) (MeOD, 600 MHz)

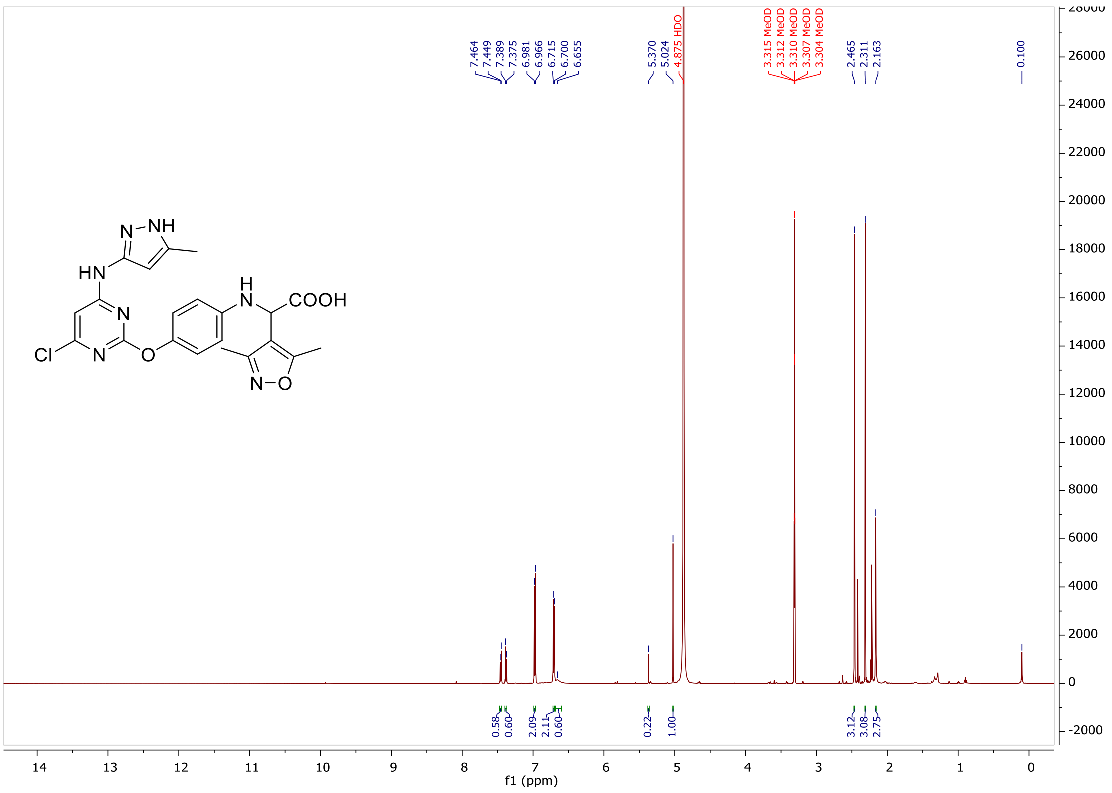

^{13}C NMR IA186 (11) (MeOD, 151 MHz)

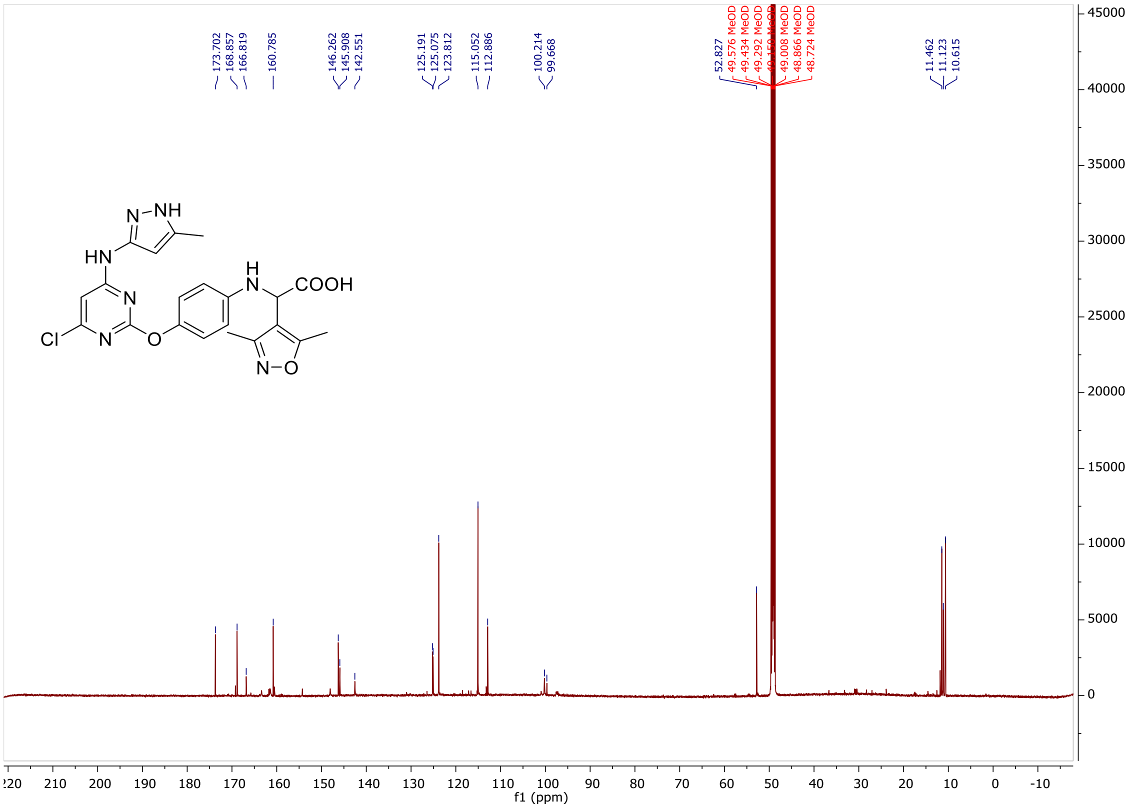

^{1}H NMR SH4 (1) (DMSO-d$_6$, 500 MHz)

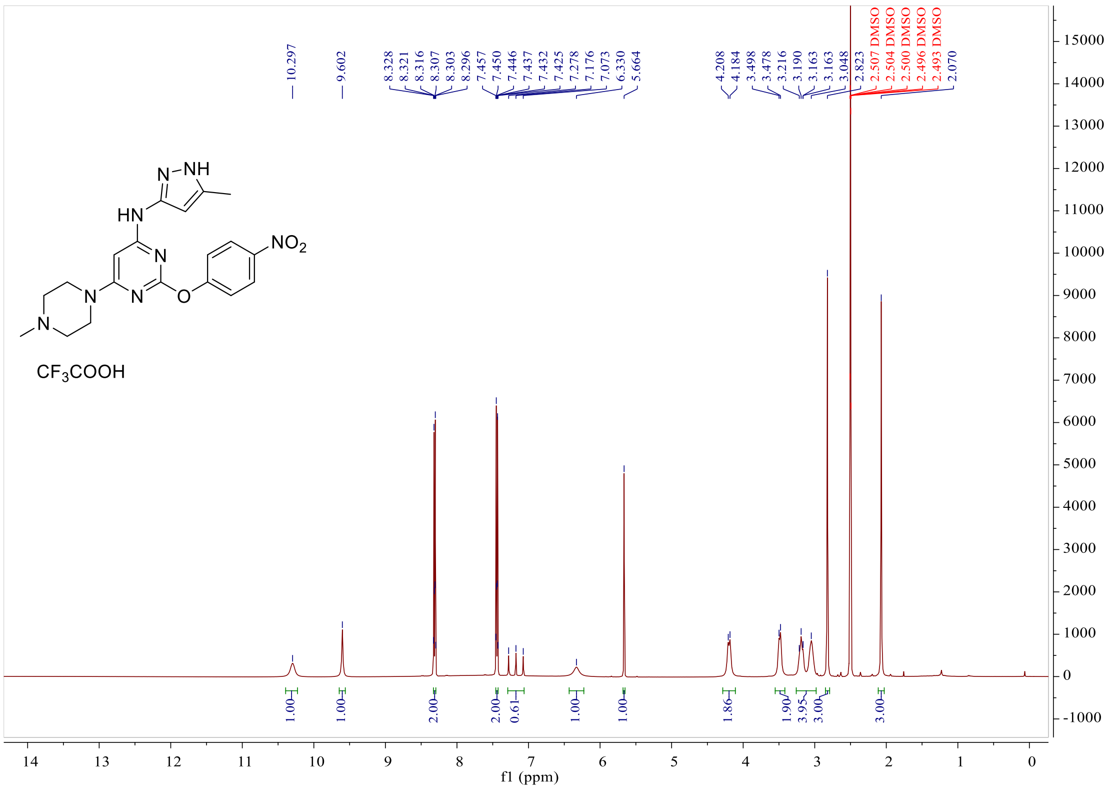

^{13}C NMR SH4 (1) (DMSO-d_6, 126 MHz)

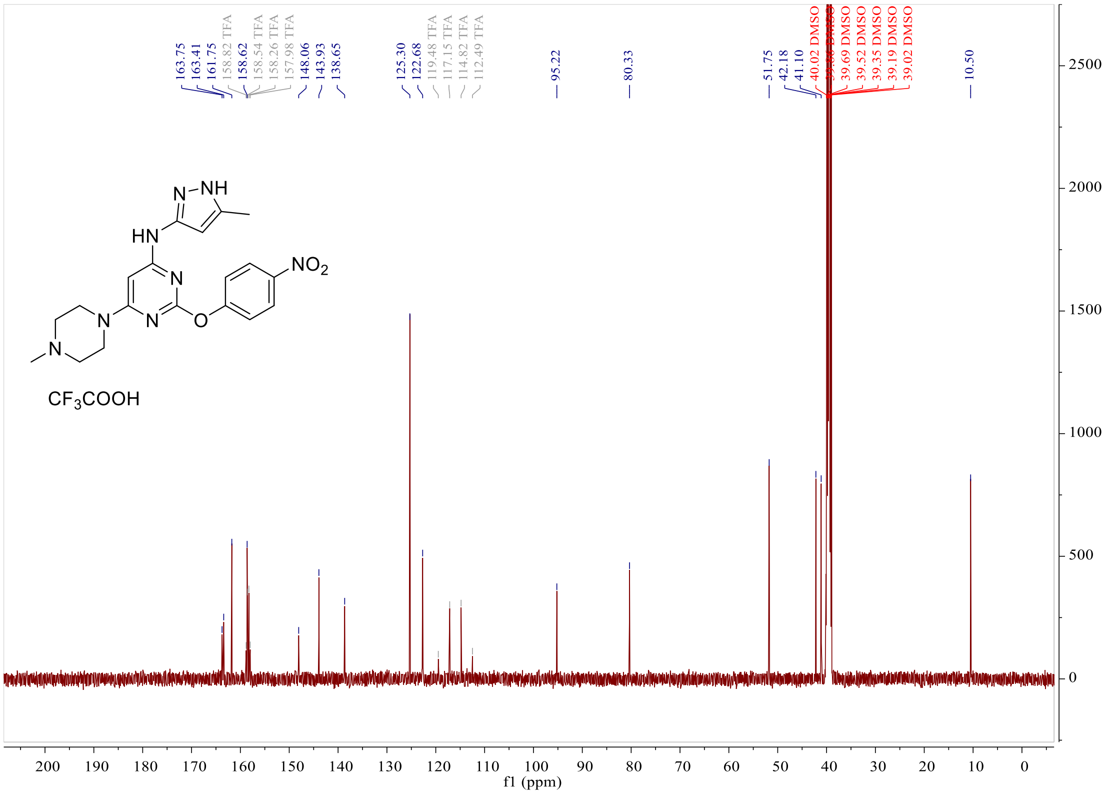

^{1}H NMR IA177 (2) (MeOD, 500 MHz)

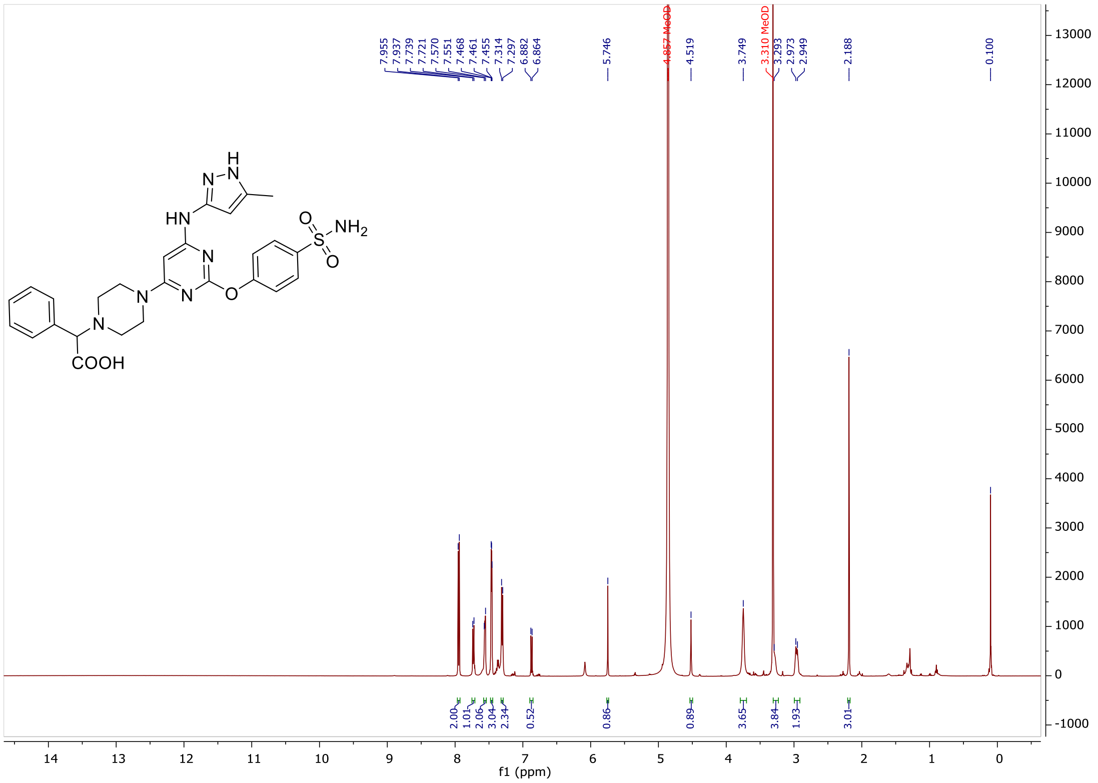

^{13}C NMR IA177 (2) (MeOD, 126 MHz)

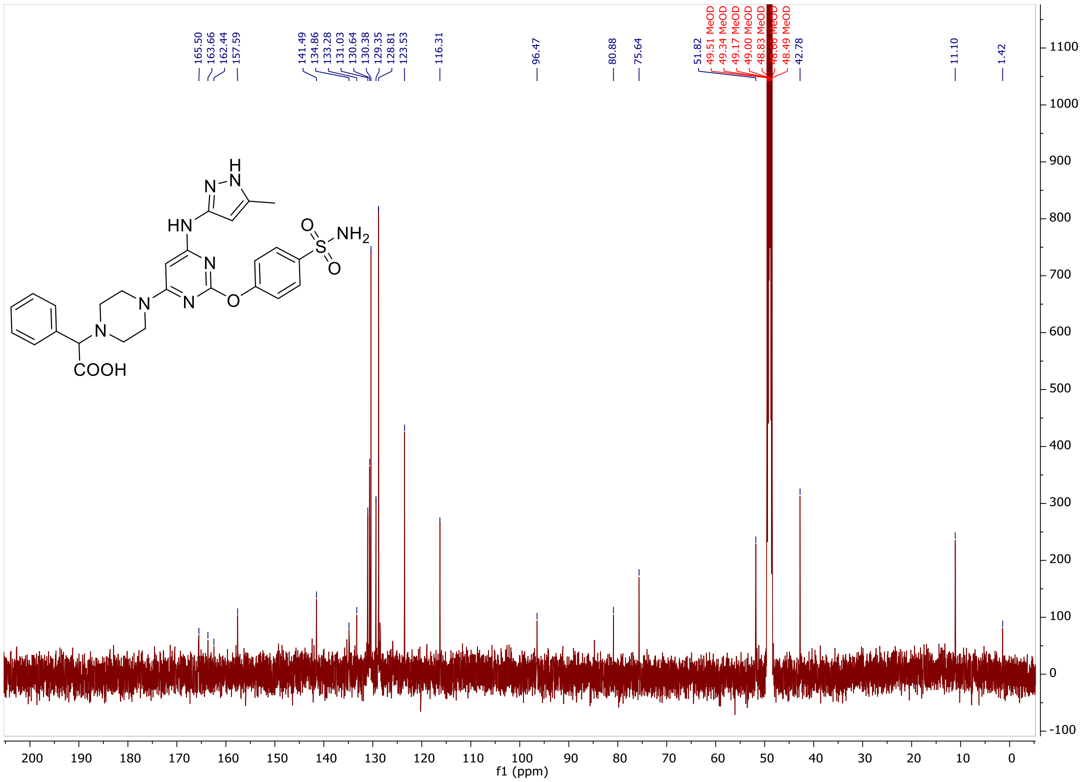

References

(1) Robke, L.; Laraia, L.; Carnero Corrales, M. A.; Konstantinidis, G.; Muroi, M.; Richters, A.; Winzker, M.; Engbring, T.; Tomassi, S.; Watanabe, N.; Osada, H.; Rauh, D.; Waldmann, H.; Wu, Y.-W.; Engel, J., Phenotypic Identification of a Novel Autophagy Inhibitor Chemotype Targeting Lipid Kinase VPS34. *Angew. Chem. Int. Ed.,* **2017**, *56*, 8153.

(2) Richters, A.; Basu, D.; Engel, J.; Ercanoglu, M. S.; Balke-Want, H.; Tesch, R.; Thomas, R. K.; Rauh, D., Identification and further development of potent TBK1 inhibitors. *ACS Chem. Biol.,* **2015**, *10*, 289.

(3) Ferri, E.; Le Thomas, A.; Wallweber, H. A.; Day, E. S.; Walters, B. T.; Kaufman, S. E.; Braun, M.-G.; Clark, K. R.; Beresini, M. H.; Mortara, K.; Chen, Y.-C. A.; Canter, B.; Phung, W.; Liu, P. S.; Lammens, A.; Ashkenazi, A.; Rudolph, J.; Wang, W., Activation of the IRE1 RNase through remodeling of the kinase front pocket by ATP-competitive ligands. *Nat. Commun.,* **2020**, *11*, 6387.
